# Hemorrhage and thrombosis in COVID-19-patients supported with extracorporeal membrane oxygenation: an international study based on the COVID-19 critical care consortium

**DOI:** 10.1186/s40560-024-00726-2

**Published:** 2024-05-06

**Authors:** Maximilian Feth, Natasha Weaver, Robert B. Fanning, Sung-Min Cho, Matthew J. Griffee, Mauro Panigada, Akram M. Zaaqoq, Ahmed Labib, Glenn J. R. Whitman, Rakesh C. Arora, Bo S. Kim, Nicole White, Jacky Y. Suen, Gianluigi Li Bassi, Giles J. Peek, Roberto Lorusso, Heidi Dalton, John F. Fraser, Jonathon P. Fanning, the COVID-19 Critical Care Consortium, Gianluigi Li Bassi, Jacky Y. Suen, Heidi J. Dalton, John Laffey, Daniel Brodie, Eddy Fan, Antoni Torres, Davide Chiumello, Alyaa Elhazmi, Carol Hodgson, Shingo Ichiba, Carlos Luna, Srinivas Murthy, Alistair Nichol, Pauline Yeung Ng, Mark Ogino, Eva Marwali, Giacomo Grasselli, Robert Bartlett, Aidan Burrell, Muhammed Elhadi, Anna Motos, Ferran Barbé, Alberto Zanella, John F. Fraser

**Affiliations:** 1 Department of Anesthesiology, Intensive Care Medicine, Emergency Medicine, and Pain Medicine, German Armed Forces Hospital Ulm, Ulm, Germany; 2 Queensland University of Technology, Brisbane, QLD Australia; 3 School of Medicine and Public Health, The University of Newcastle, New South Wales, Australia; 4 St. Vincent’s Hospital, Melbourne, VIC Australia; 5 Faculty of Medicine, University of Melbourne, Victoria, Australia; 6 Division of Cardiac Surgery, Department of Surgery, Johns Hopkins School of Medicine, Baltimore, MD USA; 7 Division of Neuroscience Critical Care, Department of Neurology and Neurosurgery, Johns Hopkins School of Medicine, Baltimore, MD USA; 8 Department of Anesthesiology and Perioperative Medicine, Sections of Critical Care and Perioperative Echocardiography, University of Utah, Salt Lake City, UT USA; 9 Anesthesiology Service, Veteran Affairs Medical Center, Salt Lake City, UT USA; 10 Department of Anesthesia, Fondazione IRCCS Ca’ Granda, Ospedale Maggiore Policlinico Di Milano, Intensive Care and Emergency, Milano, Lombardia Italy; 11 Department of Anaesthesiology, Division of Critical Care Medicine, University of Virginia, Charlottesville, VA USA; 12 Medical Intensive Care Unit, Department of Medicine, Hamad General Hospital, Hamad Medical Corporation, Doha, Qatar; 13 Harrington Heart & Vascular Institute, University Hospitals Cleveland Medical Center, Cleveland, OH USA; 14 Case Western Reserve University School of Medicine, Cleveland, OH USA; 15 Critical Care Research Group, Level 3, Clinical Sciences Building, The Prince Charles Hospital, ChermsideBrisbane, QLD 4032 Australia; 16 Faculty of Medicine, University of Queensland, Brisbane, Australia; 17 Intensive Care Unit, St Andrew’s War Memorial Hospital, UnitingCare Health, Spring Hill, QLD Australia; 18 Intensive Care Unit, The Wesley Hospital, UnitingCare Health, Auchenflower, QLD Australia; 19 Institute for Molecular Bioscience, The University of Queensland, St Lucia, QLD Australia; 20 Congenital Heart Centre, University of Florida, Gainesville, FL USA; 21 Cardiothoracic Surgery Department, Heart and Vascular Centre, Maastricht University Medical Centre, and Cardiovascular Research Institute Maastricht, Maastricht, Netherlands; 22 Heart and Vascular Institute, Inova Fairfax Hospital, Falls Church, VA USA; 23 Nuffield Department of Population Health, University of Oxford, Oxford, UK; 24 The George Institute for Global Health, Sydney, NSW Australia

**Keywords:** Coagulation disorders, COVID-19, Extracorporeal membrane oxygenation, Bleeding events, Thrombotic events

## Abstract

**Background:**

Extracorporeal membrane oxygenation (ECMO) is a rescue therapy in patients with severe acute respiratory distress syndrome (ARDS) secondary to COVID-19. While bleeding and thrombosis complicate ECMO, these events may also occur secondary to COVID-19. Data regarding bleeding and thrombotic events in COVID-19 patients on ECMO are sparse.

**Methods:**

Using the COVID-19 Critical Care Consortium database, we conducted a retrospective analysis on adult patients with severe COVID-19 requiring ECMO, including centers globally from 01/2020 to 06/2022, to determine the risk of ICU mortality associated with the occurrence of bleeding and clotting disorders.

**Results:**

Among 1,248 COVID-19 patients receiving ECMO support in the registry, coagulation complications were reported in 469 cases (38%), among whom 252 (54%) experienced hemorrhagic complications, 165 (35%) thrombotic complications, and 52 (11%) both. The hazard ratio (HR) for Intensive Care Unit mortality was higher in those with hemorrhagic-only complications than those with neither complication (adjusted HR = 1.60, 95% CI 1.28–1.99, *p* < 0.001). Death was reported in 617 of the 1248 (49.4%) with multiorgan failure (*n* = 257 of 617 [42%]), followed by respiratory failure (*n* = 130 of 617 [21%]) and septic shock [*n* = 55 of 617 (8.9%)] the leading causes.

**Conclusions:**

Coagulation disorders are frequent in COVID-19 ARDS patients receiving ECMO. Bleeding events contribute substantially to mortality in this cohort. However, this risk may be lower than previously reported in single-nation studies or early case reports.

*Trial registration* ACTRN12620000421932 (https://covid19.cochrane.org/studies/crs-13513201).

**Supplementary Information:**

The online version contains supplementary material available at 10.1186/s40560-024-00726-2.

## Clinical Perspective

- Coagulation disorders such as thrombotic or hemorrhagic events are frequent in COVID-19 ARDS patients receiving ECMO.
- While older age, pre-existing cardiac disease, and diabetes were independently associated with bleeding, prone positioning and a longer time from admission to ECMO were associated with a higher percentage of thrombotic events.
- A longer duration of ECMO was linked to an increased rate of combined hemorrhagic and thrombotic events.

**Supplementary Information:**

The online version contains supplementary material available at 10.1186/s40560-024-00726-2.

## Background

Extracorporeal membrane oxygenation (ECMO) is a cardiopulmonary support technique that can be lifesaving in patients suffering from severe respiratory and/or circulatory failure [1–3]. However, ECMO exposes patients to complications such as bleeding and thrombosis [4–6]. Coagulation disorders in critically ill patients supported with ECMO result from a complex interplay between the underlying illness and both ECMO-related (e.g., shear stress, artificial circuit surface–blood interaction) and iatrogenic factors (e.g., systemic anticoagulation) [7–9]. These complications are associated with increased morbidity and mortality [5, 10]. However, the mechanisms behind coagulation disorders during ECMO are not yet fully understood, and prevention strategies are lacking.

Severe acute respiratory syndrome coronavirus 2 (SARS-CoV-2), the virus causing coronavirus disease-2019 (COVID-19), can result in acute respiratory distress syndrome (ARDS) requiring intensive care unit (ICU) admission and advanced respiratory failure management [11, 12]. Despite optimal medical management, including mechanical ventilation and prone positioning, mortality and morbidity rates due to refractory respiratory failure among these patients are high [13, 14]. A rescue therapy in these patients is ECMO [15, 16]. The mechanisms and clinical implications of thrombotic and hemorrhagic events in COVID-19 patients supported with ECMO are areas of ongoing research. This study aimed to define the global frequency, outcomes of, and risk factors for thrombotic and hemorrhagic disorders in COVID-19 patients with refractory ARDS supported with ECMO.

## Methods

All data for this study were extracted from the global COVID-19 Critical Care Consortium (CCCC) prospective database, which was established to collect and analyze data on patients admitted to intensive care units for the treatment of severe COVID-19 [17]. The rationale and design have been previously published (Trial registration ACTRN12620000421932) [17]. Institutional Review Board (IRB) approval was obtained for each participating institution. A waiver of informed consent was granted for all patients. Additional file 1: Table S1 summarizes all the recruiting sites, including IRB approvals, contributors, and collaborators.

The CCCC database was examined for patients referred to the ICUs of 229 collaborating institutions spanning 32 countries, from January 1, 2020, through June 30, 2022. Patients who satisfied all the following criteria were entered into the registry: (1) age ≥ 16 years; (2) COVID-19 pneumonia with laboratory confirmation (real-time PCR and/ or next-generation sequencing); and (3) admission to ICU due to severe COVID-19 pneumonia. Patients admitted to critical care for conditions unrelated to COVID-19 were excluded.

Data were collected from ICU admission to either in-hospital death or hospital discharge. Data collection followed guidelines for the International Severe Acute Respiratory IncideNce sTudy of Severe Acute and Emerging Infection Consortium (ISARIC), Short-Period Incidence Study for Severe Acute Respiratory Infection (SPRINT-SARI), and the CCCC. All data obtained were de-identified and stored at a Research Electronic Data Capture (REDCap) database hosted at one of the following institutions: Oxford University, United Kingdom; University College Dublin, Ireland; or Monash University, Australia.

According to the ISARIC and the Extracorporeal Membrane Oxygenation for 2019 novel Coronavirus Acute Respiratory Distress Disease (ECMOCARD study) case report forms (CRF), adverse coagulation events included (1) thrombotic events including ischemic stroke, myocardial ischemia, myocardial infarction, deep vein thrombosis (DVT), and pulmonary embolism (PE); (2) hemorrhagic events were classified according to the bleeding site or the two predominant bleeding sources, in cases involving multiple bleeding sites; and (3) disseminated intravascular coagulation (DIC). Adverse coagulation events were diagnosed by treating physicians. The study focused on the following four patient groups treated with ECMO: (1) patients without hemorrhage or thrombosis (controls); (2) patients with both a hemorrhagic and thrombotic event; (3) patients with a hemorrhagic event only; and (4) patients with a thrombotic event only.

The study's primary outcome was mortality in COVID-19 patients supported with ECMO who suffered thrombotic and bleeding events. Secondary outcomes were the incidence of thrombotic and bleeding complications and the duration of ICU requirement (days). Additionally, we investigated risk factors for hemorrhagic or thrombotic events in COVID-19 patients on ECMO. Laboratory assessments were obtained according to the CRFs. ‘First value’ refers to a specific parameter's first recorded value in the CRFs. Minimum and maximum values are the minimum/maximum level of a parameter from enrolling in the study throughout the follow-up period.

### Statistical analysis

The study cohort was limited to patients who were treated with ECMO. Patients without thrombotic or hemorrhagic complications were compared to the following subgroups: patients with a hemorrhagic event only, a thrombotic event only, or a combination of hemorrhagic and thrombotic events. Demographic characteristics, medical history, critical care treatment, and outcomes were described and checked for missing data (Additional file 1: Table S2). Continuous data were summarized as mean with standard deviation or median with interquartile range. Categorical variables were summarized as frequency count and percentage. Differences between groups were evaluated using Pearson's chi-squared test for categorical variables and the Wilcoxon–Mann–Whitney *U* test for continuous variables.

Survival analysis was used to estimate the effect of coagulation complications (combined and for thrombotic and hemorrhagic complications separately) on the time between ICU admission and mortality. The survival analysis cohort was limited to patients with non-missing discharge status and a valid ICU discharge date. The effect of coagulation complications on the instantaneous mortality hazard was estimated using Cox regression, assuming patients ‘discharged alive’ (alive, home, palliative care, hospitalized, or transferred to another facility) were censored independently. The proportional hazards assumption was verified with log–log plots and a test of Schoenfeld residuals. Parametric Weibull regression also was performed as a sensitivity analysis. Each survival analysis method was used to produce crude estimates and estimates adjusted a priori for patient age, sex, body mass index (BMI), and country of hospitalization. Due to a large proportion of missing BMI data, all analyses were repeated without adjusting for BMI. Regression results were presented as hazard ratios with 95% confidence intervals and p values.

Analysis was performed in SAS 9.4 (SAS Institute Inc., Cary, NC, USA), apart from survival analyses performed in Stata 15 (StataCorp, College Station, TX, USA).

## Results

**Fig. 1:**
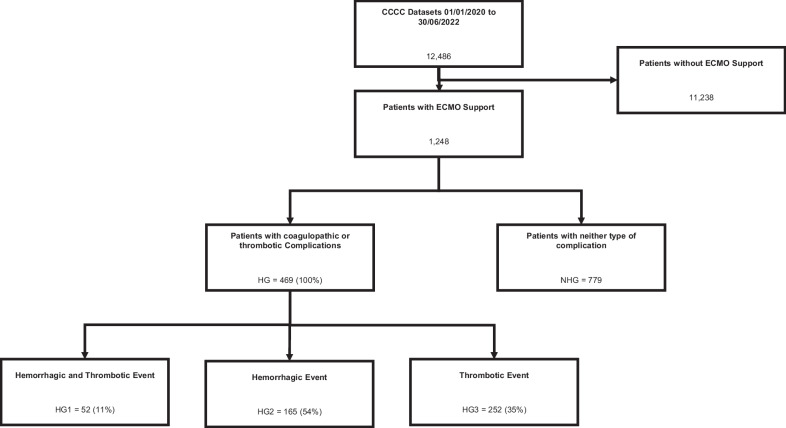
Study Cohort, Flow Chart. CCCC Covid Critical Care Consortium, ECMO extracorporeal membrane oxygenation

During the study period, 1,248 patients receiving VV- or VA-ECMO support due to COVID-19-related critical illness were included in the CCCC database. Table 1 summarizes baseline patient characteristics, including pre-existing health and management conditions. A hemorrhagic or thrombotic event was documented in 469 (38%). Among these 469 patients, 52 (11%) experienced at least one hemorrhagic and one thrombotic complication, while 252 (54%) patients experienced a hemorrhagic event only and 165 (35%) a thrombotic event only (Fig. 1),

**Table 1:** Baseline patient characteristics with accompanying univariate analysis

Characteristic	Class or Statistic	Neither (*n* = 779)	Both (*n* = 52)	Hemorrhagic only (*n* = 252)	Thrombotic only (*n* = 165)	Both vs. Neither	Hemorrhagic only vs. Neither	Thrombotic only vs. Neither
Age (years)	median (Q1, Q3)	50.0 (40.0, 58.0)	55.0 (42.5, 62.0)	52.5 (43.0, 60.0)	49.0 (40.0, 58.0)	0.0697	**0.0104**	0.8369
Body mass index (kg/m^2^)	median (Q1, Q3)	30.4 (27.2, 34.9)	28.8 (25.2, 31.1)	29.9 (26.3, 34.0)	31.9 (27.3, 36.0)	0.2094	0.6844	0.1444
Sex	Female	235 (30%)	18 (35%)	72 (29%)	53 (32%)	0.500	0.630	0.620
	Male	544 (70%)	34 (65%)	180 (71%)	112 (68%)			
Ethnicity	White	166 (28%)	22 (46%)	117 (49%)	57 (43%)	**0.015**	** < 0.001**	** < 0.001**
	Black	53 (8.9%)	7 (15%)	18 (7.6%)	11 (8.3%)			
	Asian	66 (11%)	4 (8.3%)	30 (13%)	12 (9.0%)			
	Hispanic	227 (29%)	9 (17%)	37 (15%)	16 (9.7%)			
	Aboriginal	9 (1.2%)	0	1 (0.4%)	2 (1.2%)			
	Other	81 (14%)	7 (15%)	35 (15%)	36 (27%)			
Chronic cardiac disease	Yes	29 (4.5%)	5 (9.8%)	25 (10%)	9 (5.7%)	0.095	**0.002**	0.544
Chronic kidney disease	Yes	33 (5.2%)	4 (7.8%)	8 (3.3%)	4 (2.5%)	0.417	0.235	0.161
Chronic neurological disorder	Yes	14 (2.3%)	2 (3.9%)	7 (2.9%)	1 (0.6%)	0.479	0.632	0.173
Chronic hematologic disorder	Yes	19 (3.2%)	0	7 (2.9%)	4 (2.6%)	0.198	0.831	0.699
Diabetes	Yes	108 (18%)	10 (20%)	62 (26%)	35 (23%)	0.724	**0.008**	0.127
Hypertension	Yes	274 (43%)	21 (41%)	102 (41%)	59 (37%)	0.791	0.630	0.172
Smoking	Never smoked	293 (48%)	26 (50%)	109 (45%)	65 (42%)	0.931	0.681	0.240
	Current smoker	103 (17%)	9 (17%)	46 (19%)	24 (15%)			
	Former smoker	216 (35%)	17 (33%)	86 (36%)	66 (43%)			
Malignant neoplasm	Yes	7 (1.2%)	1 (2.0%)	4 (1.7%)	1 (0.6%)	0.620	0.567	0.564
SOFA score	Median (Q1, Q3)	7.0 (4.0, 10.0)	7.5 (4.0, 10.0)	7.0 (5.0, 10.0)	8.0 (5.0, 10.0)	0.8473	0.5001	0.6218
APACHE II score	Median (Q1, Q3)	17.0 (10.0, 23.0)	22.0 (16.0, 27.0)	17.5 (11.5, 23.0)	20.0 (15.5, 23.5)	0.1378	0.9893	0.0471

Statistically significant p-values for intergroup differences are presented in bold

*SOFA*, Sequential Organ Failure Assessment; *APACHE*, Acute Physiology and Chronic Health Evaluation

### Outcomes and causes of death

**Fig. 2:**
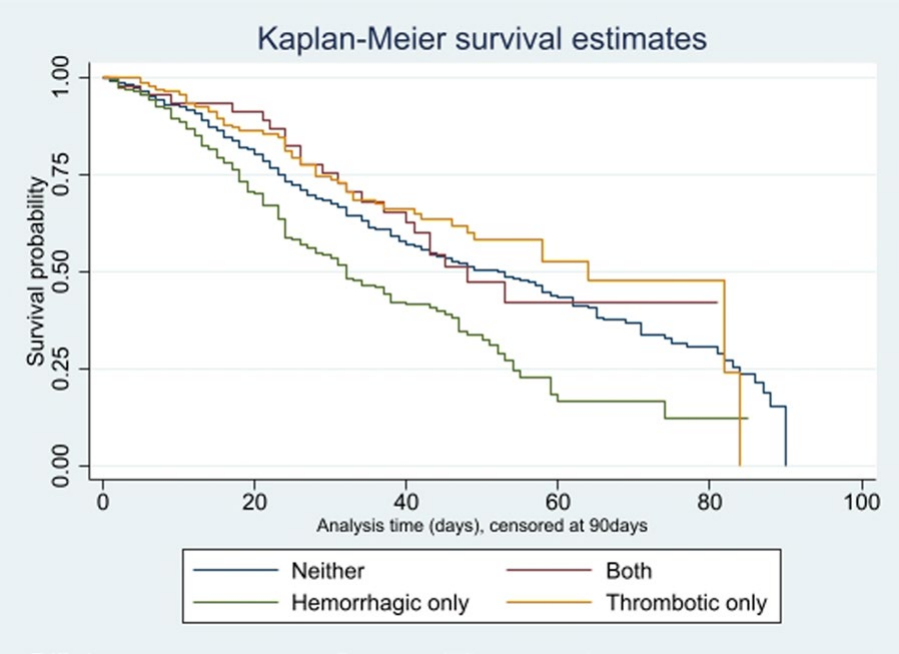
Kaplan–Meier Curve comparing patients with a thrombotic event, a hemorrhagic event, both events and neither event. NB Log rank test for equality of survivor functions *p* < 0.0001

The adjusted hazard ratio (HR) for ICU mortality was higher among patients who experienced only a hemorrhagic complication than in patients who had neither type of complication (adjusted HR = 1.60, 95% CI 1.28–1.99, *p* < 0.001, Table 2). No statistically significant differences in ICU mortality were observed in patients with both types of complication (adjusted HR = 1.02, 95% CI 0.67–1.57, *p* = 0.918) or thrombotic events only (adjusted HR 0.79, 95% CI 0.59–1.05, *p* = 0.103) relative to patients with neither type of complication. Figure 2 depicts the survival of COVID-19 patients supported with ECMO over time in the four study groups.

**Table 2:** Hazard Ratios for ICU mortality throughout the study groups among all patients enrolled as well as among all patients supported with venovenous ECMO. Presented are unadjusted and adjusted (patient age, sex, country) Hazard Ratios

Study group	Unadjusted HR (95% CI)	Unadjusted *p* value	Adjusted HR (95% CI)	Adjusted *p* value
All patients enrolled (*n* = 1119 eligible patients)				
Neither type of complication	Reference group		Reference group	
Both types of complications	0.99 (0.65, 1.50)	0.948	1.02 (0.67, 1.57)	0.918
Hemorrhagic complication only	1.55 (1.27, 1.90)	** < 0.001**	1.60 (1.28, 1.99)	** < 0.001**
Thrombotic complication only	0.82 (0.62, 1.09)	0.173	0.79 (0.59, 1.05)	0.103
Patients supported with vvECMO (*n* = 760 eligible patients)				
Neither type of complication	reference group		reference group	
Both types of complications	0.76 (0.47, 1.23)	0.262	0.80 (0.49, 1.31)	0.379
Hemorrhagic complication only	1.43 (1.13, 1.81)	**0.003**	1.42 (1.10, 1.84)	**0.008**
Thrombotic complication only	0.67 (0.48, 0.92)	**0.013**	0.64 (0.46, 0.89)	**0.008**

Statistically significant p-values for intergroup differences are presented in bold

*HR* Hazard Ratio**;** *CI* Confidence Interval

The length of stay (days) within the ICU was longer for patients with both types of complication (42.0 days, 27.5–52.5, *p* = 0.009) and for those with a thrombotic event only (37.0 days, 24.0–57.0, *p* = 0.010) than in patients with neither type of complication (30.0 days, 17.0–52.0). Hospital length of stay was longer for those with both types of complication (45.0 days, 29.0–72.0, *p* = 0.017) and those with thrombotic events (44.0 days, 26.0–69.0, *p* = 0.003), but shorter among those with hemorrhagic events (28.0 days, 14.0–50.0, *p* = 0.001) compared to patients with neither type of complication (35.0 days, 19.0–59.0).

Overall, 617 of 1248 patients (49.4%) died in the ICU. The leading cause of death was multiorgan failure (257, 42%), followed by respiratory failure (130, 21%) and septic shock (55, 8.9%) (Table 3).

**Table 3:** Cause of death for patients requiring ECMO with ICU mortality

Cause of death	Neither (*n* = 358)	Both (*n* = 27)	Hemorrhagic only (*n* = 160)	Thrombotic only (*n* = 72)	Total deaths (*n* = 617)
Multi-organ failure	146 (41%)	9 (33%)	59 (37%)	43 (60%)	257 (42%)
Respiratory failure	72 (20%)	6 (22%)	33 (21%)	19 (26%)	130 (21%)
Septic shock	30 (8.4%)	3 (11%)	22 (14%)	0	55 (8.9%)
Cardiac failure	29 (8.1%)	2 (7.4%)	6 (3.8%)	5 (6.9%)	42 (6.8%)
Unknown*	33 (9.2%)	0	3 (1.9%)	2 (2.8%)	38 (6.2%)
“Other”	23 (6.4%)	1 (3.7%)	10 (6.3%)	3 (4.2%)	37 (6.0%)
Cerebrovascular accident	15 (4.2%)	4 (15%)	17 (11%)	0	36 (5.8%)
Hemorrhagic shock	8 (2.2%)	1 (3.7%)	8 (5.0%)	0	17 (2.8%)
Cardiovascular event	1 (0.3%)	0	2 (1.3%)	0	3 (0.5%)
Liver failure	1 (0.3%)	1 (3.7%)	0	0	2 (0.3%)

^*^This table refers to cases with no cause of death mentioned as “unknown” including 1 case with the cause of death mentioned as “not applicable”. Additional causes of death were summarized as “other”

### Coagulation complications (Table 4)

**Table 4:** Frequency of thrombosis and hemorrhagic complications in ECMO patients

Complication	ECMO Cohort (*n* = 1248)
All coagulopathic or thrombotic = complications	469 (38%)
Thrombotic	217 (17% of ECMO)
Pulmonary embolism	86 (40%)
Deep vein thrombosis	77 (35%)
Myocardial infarction/cardiac ischemia	38 (18%)
Ischemic Stroke or cerebrovascular accident	9 (4.1%)
Other thromboembolism	41 (19%)
Hemorrhagic	304 (24% of ECMO)
*Hemorrhagic complications, site(s):*	
Lungs	52 (17%)
Gastrointestinal	112 (37%)
Genitourinary	20 (6.6%)
Skin and soft tissue	48 (16%)
CNS/hemorrhagic stroke	59 (19%)
Cardiac	3 (1.0%)
ECMO cannula site	69 (23%)
Iliopsoas	7 (2.3%)
Unknown site	37 (12%)
Other	5 (1.6% of ECMO)

*CNS* central nervous system; *ECMO* extracorporeal membrane oxygenation

Thrombotic complications were documented in 217 (17.4%) of the 1248 patients with pulmonary embolism being the most common (*n* = 86 or 39.6%). Hemorrhagic complications occurred in 304 (24%) of all patients with the most common source being gastrointestinal (112, 36.8%). Note that bleeding severity was not part of the case report forms and, therefore, cannot be commented on.

The most common anticoagulation prophylaxis method was unfractionated heparin (UFH), followed by low molecular weight heparin (LMWH). Other anticoagulation strategies were rarely used (Table 5). Table 6 summarizes laboratory assessments.

**Table 5:** Clinical and anticoagulation management with accompanying univariate analysis

Characteristic	Class or statistic	Neither (*n* = 779)	Both (*n* = 52)	Hemorrhagic only (*n* = 252)	Thrombotic only (*n* = 165)	Both vs. neither	Hemorrhagic only vs. neither	Thrombotic only vs. neither
Any invasive ventilation	Yes	776 (99.6%)	51 (98%)	252 (100.0%)	165 (100.0%)	0.121	0.324	0.425
Mechanical ventilation	Yes	576 (96%)	50 (96%)	241 (99%)	157 (96%)	0.902	0.071	0.920
Mechanical ventilation (days)	median (Q1, Q3)	26.0 (14.0, 46.0)	39.0 (28.0, 51.0)	26.5 (15.0, 43.5)	35.0 (19.0, 52.0)	**0.0022**	0.9107	**0.0051**
Time from admission to mechanical ventilation (days)	median (Q1, Q3)	1.0 (0.0, 5.0)	3.0 (0.0, 7.0)	1.0 (0.0, 5.0)	1.0 (0.0, 5.0)	0.0864	0.8608	0.8986
Prone positioning (mechanical ventilation)	Yes	345 (69%)	35 (67%)	170 (71%)	111 (81%)	0.786	0.581	**0.006**
Prone positioning (before ECMO)	Yes	279 (69%)	33 (66%)	136 (64%)	90 (78%)	0.642	0.175	0.080
Inhaled nitric oxide	Yes	110 (22%)	15 (29%)	62 (26%)	34 (24%)	0.245	0.306	0.607
Neuromuscular blockade (before ECMO)	Yes	333 (79%)	38 (78%)	164 (78%)	86 (74%)	0.802	0.856	0.253
Tracheostomy	Yes	289 (50%)	36 (71%)	130 (54%)	112 (69%)	**0.004**	0.273	** < 0.001**
ECMO (days)	median (Q1, Q3)	19.0 (9.0, 34.0)	26.5 (18.5, 36.0)	16.0 (8.0, 30.0)	18.0 (9.0, 34.0)	**0.0146**	0.0750	0.6673
Time from admission to ECMO (days)	median (Q1, Q3)	0.0 (0.0, 6.0)	2.0 (0.0, 9.0)	1.0 (0.0, 6.0)	1.0 (0.0, 7.0)	0.0595	0.1036	**0.0425**
Vasopressor use	Yes	483 (85%)	47 (90%)	225 (92%)	144 (89%)	0.261	**0.003**	0.121
Transfusion—any blood product	Yes	354 (45%)	37 (71%)	182 (72%)	95 (58%)	** < 0.001**	** < 0.001**	**0.005**
Transfusion—red blood cells	Yes	248 (32%)	35 (67%)	179 (71%)	71 (43%)	** < 0.001**	** < 0.001**	**0.006**
Transfusion—platelets	Yes	136 (17%)	17 (33%)	60 (24%)	33 (20%)	**0.006**	**0.026**	0.439
Transfusion—fresh frozen plasma	Yes	45 (5.8%)	11 (21%)	53 (21%)	8 (4.8%)	** < 0.001**	** < 0.001**	0.638
Transfusion—cryoprecipitates	Yes	23 (3.0%)	7 (13%)	16 (6.3%)	3 (1.8%)	** < 0.001**	**0.014**	0.419
ICU length of stay (days)	Median (Q1, Q3)	30.0 (17.0, 52.0)	42.0 (27.5, 52.5)	27.0 (17.0, 47.0)	37.0 (24.0, 57.0)	**0.0090**	0.2556	**0.0096**
Hospital length of stay (days)	Median (Q1, Q3)	35.0 (19.0, 59.0)	45.0 (29.0, 72.0)	28.0 (14.0, 50.0)	44.0 (26.0, 69.0)	**0.0167**	**0.0011**	**0.0031**
Anticoagulation therapy	Yes	300 (98%)	36 (100%)	119 (98%)	104 (99%)	0.439	0.573	0.613
Anticoagulation medication	Direct Oral Anticoagulant (DOAC)	12 (4.4%)	0	2 (1.8%)	4 (4.2%)	0.356	0.343	0.967
	Enoxaparin/Low molecular weight heparin (LMWH)	77 (28%)	12 (35%)	37 (33%)	28 (29%)			
	Unfractionated heparin (UFH)	185 (68%)	22 (65%)	74 (65%)	63 (66%)			
Anticoagulation route	Subcutaneous	91 (12%)	17 (33%)	40 (16%)	38 (23%)	** < 0.001**	0.086	** < 0.001**

Statistically significant p-values for intergroup differences are presented in bold

*ECMO* extracorporeal membrane oxygenation

**Table 6:** Laboratory evaluations. First values are the first values given in the CRFs for a specific parameter. Minimum and maximum values are the minimum/maximum level of a parameter from inclusion in the study throughout the follow-up period. PT, prothrombin time, INR, international normalized ratio

Characteristic	Class or Statistic			Neither (*n* = 779)		Both (*n* = 52)		Hemorrhagic only (*n* = 252)		Thrombotic only (*n* = 165)	*p* value
			*N*	Mean (SD)	*N*	Mean (SD)	*N*	Mean (SD)	*N*	Mean (SD)	
d-Dimer (ng/mL)	First		452	373 (1966.83)	40	342.88 (1660.1)	173	89.41 (49604)	123	107.78 (720.3)	** < 0.001**
	Maximum		452	1047.25 (4238.86)	40	2815.21	173	414.81 (2014.83)	123	178.45 (906.23)	** < 0.001**
Troponin (ng/mL)	First		189	14.78 (78.59)	16	19.25 (49.76)	70	12.53 (43.19)	61	1311.21	** < 0.001**
	Maximum		189	16.66 (79.33)	16	19.29 (49.74)	70	14.38 (45.7)	61	1312.23	** < 0.001**
Hemoglobin (g/dL)	First		579	11.55 (304)	48	11.74 (284)	232	11.47 (2.64)	151	11.55 (2.59)	**0.022**
	Minimum		579	8.17 (2.85)	48	7.81 (1.62)	232	7.69 (2.03)	151	7.87 (1.97)	** < 0.001**
Platelet Count (10^3^/μL)	First		580	226.83 (119.07)	48	211.84 (100.47)	232	223.97 (111.69)	151	243.18 (131)	0.069
	Minimum		580	131.48 (86.66)	48	96.12 (59.91)	232	107.54 (79.89)	151	133.93 (94.54)	**0.002**
	Maximum		580	312.14 (160.59)	48	273.09 (119.32)	232	295.19 (141 05)	151	352,08 (180.04)	**0.001**
PT (seconds)	First		409	16.38 (13.77)	34	20.29 (18.73)	141	19.84 (17.93)	117	18.07 (15.71)	** < 0.001**
	Maximum		409	20.88 (17.95)	34	24.51 (21.29)	141	26.06 (2346)	117	22.53 (17.62)	** < 0.001**
Fibrinogen (mg/dL)	First		243	623.08 (922.01)	30	474.35 (209.11)	120	501.02 (222.06)	73	535.78 (231.67)	** < 0.001**
	Minimum		243	465.17 (925.21)	30	350.61 (201.78)	120	357.49 (194.9)	73	380.87 (215.88)	** < 0.001**

Statistically significant p-values for intergroup differences are presented in bold

### Advanced ARDS management and ECMO

Clinical management of COVID-19 patients supported with ECMO is shown in Table 5, while Additional file 1: Table S3 provides ECMO specific data. Prone positioning during mechanical ventilation was more common in patients with thrombotic events than in controls (111, 81% vs. 354, 69%, *p* = 0.006). Furthermore, in patients with both types of complication (36/52, 71%, *p* = 0.004) as well as in patients with just a thrombotic event (112/165, 69%, *p* < 0.001), tracheostomy was more commonly performed than in controls (289/779, 50%).

Most patients received venovenous (864, 93.8%) rather than venoarterial ECMO (57, 6.2%). Time to admission for ECMO was statistically longer for patients with thrombotic events than in controls (*p* = 0.043). Duration of ECMO support also was statistically longer among patients with both complication types (*p* = 0.015). Maximum and mean daily ECMO blood flow was significantly less in patients with only thrombotic events than in patients with either hemorrhage events only, as well as among those with either, both, or neither type of complication (maximum daily blood flow *p* = 0.010, mean daily blood flow rate *p* = 0.015). However, there was no statistically significant difference in mean daily blood flow rates once adjusted for patient body weight. Circuit changes were most frequent in patients with both types of complications (26%), followed by those with hemorrhage complications (22%) and those with neither type of complication (16%). The incidence of any circuit change was the least frequent in patients with a thrombotic event (12%).

When considering venovenous ECMO only, we found a higher adjusted HR for ICU mortality for patients with hemorrhagic complications (adjusted HR 1.42, 95% CI 1.10–1.84, *p* = 0.008) compared to those without either type of complication. In contrast to the entire cohort, we observed a statistically significant reduction in HR for ICU mortality for venovenous ECMO patients with thrombotic complications only (HR, 0.64, 95% CI 0.46–0.89, *p* = 0.008) compared to venovenous ECMO patients without either type of complication (Table 2).

### International comparison

This study involved participants mainly from the United States (*n* = 354), Colombia (*n* = 215), Spain (*n* = 140), Italy (*n* = 140), Kuwait (*n* = 126) and Australia (*n* = 12). Mortality was highest in Italy (64%), lowest in Australia (33%), and comparable (47–56%) among the other countries. However, ICU length of stay was not significantly different between regions. Table 7 summarizes further parameters by the host nation.

**Table 7:** Differences in outcomes and demographics by country of submitting center. This table depicts a majority of all patients recruited in 6 leading countries

Characteristic	Category or statistic	United States (*n* = 354)	Colombia (*n* = 215)	Spain (*n* = 140)	Italy (*n* = 140)	Kuwait (*n* = 126)	Australia (*n* = 12)
Age (years)	Median (Q1, Q3)	49.0 (38.0, 57.0)	47.0 (38.0, 55.0)	55.0 (47.0, 61.0)	54.0 (48.0, 60.0)	42.0 (35.0, 50.0)	49.0 (43.0, 61.5)
Body mass index (kg/m2)	Median (Q1, Q3)	32.9 (28.6, 38.6)	29.1 (27.0, 32.7)	29.4 (25.9, 32.7)	29.3 (26.3, 32.7)	30.9 (27.7, 35.5)	27.7 (22.1, 33.9)
ICU length of stay (days)	Median (Q1, Q3)	31.0 (19.0, 47.0)	38.0 (16.0, 66.0)	25.5 (13.0, 43.0)	31.0 (18.0, 47.0)	35.5 (21.0, 52.0)	28.0 (22.5, 45.5)
Hospital length of stay (days)	Median (Q1, Q3)	35.0 (20.0, 54.0)	42.0 (19.0, 71.0)	30.0 (16.0, 52.0)	34.0 (17.0, 58.0)	47.0 (24.0, 65.0)	51.5 (37.5, 114.0)
Sex	Female	132 (37%)	60 (28%)	28 (20%)	28 (20%)	48 (38%)	7 (58%)
	Male	222 (63%)	155 (72%)	112 (80%)	112 (80%)	78 (62%)	5 (42%)
Ethnicity	White	120 (38%)		17 (85%)	93 (89%)	1 (0.8%)	3 (30%)
	Black	82 (26%)			2 (1.9%)	2 (1.6%)	
	Asian	9 (2.8%)			2 (1.9%)	36 (29%)	3 (30%)
	Hispanic, aboriginal	57 (18%)	215 (100.0%)	1 (5.0%)	6 (5.7%)		
	Other	50 (16%)		2 (10%)	2 (1.9%)	84 (68%)	4 (40%)
Comorbidity obesity	No	169 (49%)	46 (53%)	83 (59%)	60 (58%)	110 (89%)	6 (50%)
	Yes	179 (51%)	41 (47%)	57 (41%)	43 (42%)	14 (11%)	6 (50%)
Discharge disposition	Discharged dead	160 (45%)	102 (47%)	67 (48%)	89 (64%)	70 (56%)	4 (33%)
	Discharged alive	110 (31%)	111 (52%)	66 (47%)	33 (24%)	26 (21%)	3 (25%)
	Hospitalization	2 (0.6%)		1 (0.7%)	11 (7.9%)	9 (7.1%)	
	Transferred to other facility	80 (23%)	2 (0.9%)	6 (4.3%)	7 (5.0%)	21 (17%)	5 (42%)
	Palliative discharge	2 (0.6%)					
Mortality at 28 days	No	260 (75%)	157 (74%)	31 (46.3%)	95 (69%)	93 (76%)	10 (91%)
	Yes	87 (25%)	55 (26%)	36 (53.7%)	43 (31%)	29 (24%)	1 (9.1%)
Mortality at 90 days	No	205 (59%)	127 (60%)	4 (5.9%)	75 (54%)	56 (46%)	8 (73%)
	Yes	142 (41%)	85 (40%)	63 (94.1%)	63 (46%)	66 (54%)	3 (27%)

## Discussion

In this international registry, we found that coagulation-related complications occurred in 38% of patients with severe COVID-19 requiring ECMO (hemorrhagic 20.2%; thrombotic 13.2%, and both < 5%). Hemorrhagic events were associated with increased mortality, whereas thrombotic events, alone or combined with hemorrhagic events, did not significantly impact mortality. In a recent study by Mansour et al., 66% of 620 critically ill COVID-19 patients receiving ECMO in France experienced coagulation disorders: 29% had bleeding, 16% thrombotic events, and 20% had both. Compared to this French cohort, our global CCCC study observed a lower incidence of bleeding and combined complications, with thrombotic events being comparable (13.2 vs. 16%). Differences in the choice of anticoagulant agent and/or the therapeutic target level might have contributed to the lower rate of bleeding events we observed in CCCC registry patients. Another potential explanation for the difference in the incidence of bleeding events might be how bleeding events were defined and captured. Nevertheless, both our study and that of Mansour et al. identified an association between coagulation disorders and increased mortality.

Within our population, those experiencing only hemorrhagic but not thrombotic event (alone or in combination) experienced a greater hazard of ICU mortality. This might be due to the high rates of mortality associated with certain types of bleeding, such as intracranial hemorrhage and severe bleeding requiring massive transfusion. Our finding of a reduced hazard of ICU mortality for patients experiencing thrombotic events contrasts with the reports of patients requiring ECMO due to non-Covid-19 conditions who undergo thrombosis. This might either be due to the differences of prothrombotic tendencies of different COVID-19 phenotypes or to the already increased risk of thrombosis resulting from prolonged critical care. Unfortunately, due to missing data, we could not adjust our survival analysis for other factors that might have contributed to mortality in this group. Therefore, though hypothesis generating, our mortality findings should be interpreted with caution.

In our cohort, multi-organ as well as respiratory failure and septic shock were the leading causes of death. This mirrors results reported by Peek et al. in 2009, who found that multi-organ failure accounted for 42% of the deaths in patients treated with ECMO [18]. Death due to hemorrhagic shock or cerebrovascular events was rare, even though bleeding was identified as a risk factor for mortality. Ischemic stroke and cerebrovascular accidents, generally considered frequent causes of permanent impairment after ECMO, occurred in nine patients in our study (4.1% among patients with a thrombotic event and 0.72% of the entire cohort), which is comparable to the incidence of stroke in a non-COVID ECMO group investigated in the EOLIA trial [2].

Our study identified several factors independently associated with coagulation disorders: older age, pre-existing cardiac disease, and diabetes were associated with bleeding events, while White ethnicity was associated with an increased risk of all coagulation disorders. Extended ECMO duration was associated with an increased incidence of bleeding but not thrombotic events, diverging from past reports in both in COVID and non-COVID patient populations. Longer mechanical ventilation was associated with both thrombotic and combined complications, but not with bleeding events alone. Both prone positioning during mechanical ventilation and longer time from admission to ECMO were associated with a higher incidence of thrombotic events. This aligns with Gebhard et al.’s 2021 study, which found extended prone positioning increased DVT risk in a small cohort [19]. These findings suggest a need for vigilance and close monitoring for thrombosis in ECMO patients undergoing prone positioning, awaiting further studies to clarify this relationship.

Subcutaneous administration of anticoagulation was associated with thrombotic complications (both combined and individual), suggesting that this route might not be suitable for preventing thrombosis in COVID-19 ECMO patients. This finding contrasts with Wiegele et al.’s single-center study, where ECMO patients treated with subcutaneous enoxaparin experienced fewer thrombotic or major bleeding events than those receiving unfractionated heparin [20].

Blood product transfusion was frequent in patients with either or both complications. Transfusion of packed red blood cells was independently associated with both forms of complication (alone or combined). However, platelets, fresh frozen plasma, and cryoprecipitate transfusions occurred more in patients with bleeding events, regardless of whether they were combined with thrombotic complications, but not in patients with only thrombotic events.

### Strengths and limitations

This study has several limitations, including missing data and the retrospective nature of data extraction. Despite using standardized case report forms to minimize variations in data reporting, data entry depended on the discretion of physicians and research staff at each participating center and consequently, data completeness was heterogeneous. In addition, variability in ECMO and critical care management across centers, coupled with the voluntary nature of site participation, may have skewed representation to those with sufficient resources to enter the data. This variability hinders the precise assessment of potentially outcome-impacting factors such as the anticoagulation practices and ECMO management protocols.

On the other hand, extensive international collaboration offers valuable insights into thrombotic and bleeding events in COVID-19 ECMO patients globally. The pandemic’s evolving nature and the consequent adaptations in patient management strategies across different COVID waves add complexity to our analysis, particularly as our data collection tools could not be updated to reflect these changes, omitting potentially significant factors like immunomodulatory treatments and vaccination impacts on thrombotic and hemorrhagic complications. Additionally, the case report forms did not define bleeding severity, which might have led to heterogeneous reporting of bleeding events.

Notably, our study found no link between thrombotic events and mortality, possibly due to the lack of a detailed thrombosis severity assessment and the inclusion of minor thrombotic events. Future research should aim for clear definitions and severity grading of hemorrhagic and thrombotic events to enhance understanding and management of these complications.

## Conclusions

In an international registry for critically ill COVID-19 patients receiving ECMO, the incidence of bleeding and thrombotic complications were high, albeit lower than previously reported. Bleeding significantly elevated mortality risk, with multi-organ failure and sepsis as the primary causes of death. Factors such as older age and White ethnicity were associated with an increased incidence of bleeding. Extended ECMO duration corresponded with higher bleeding rates but did not affect the occurrence of thrombotic events.

### Supplementary Information

**Additional file 1**. Supplemental Material.

## Data Availability

The datasets used and/ or analyzed during the current study are available from the corresponding author in reasonable request.

## Abbreviations

ARDS: Acute respiratory distress syndrome
BMI: Body mass index
CCCC: COVID critical care consortium
DIC: Disseminated intravascular coagulation
DVT: Deep vein thrombosis
ECMO: Extracorporeal membrane oxygenation
ICU: Intensive care unit
ISARIC: International severe acute respiratory and emerging infection consortium
LMWH: Low molecular weight heparin
PCR: Polymerase chain reaction
PE: Pulmonary embolism
REDCap: Research electronic data capture
SARS-CoV2: Severe acute respiratory syndrome coronavirus 2
SPRINT-SARI: Short-period incidence study of severe acute respiratory infection
UFH: Unfractionated heparin
