# Supplementary material for: Hemorrhage and thrombosis in COVID-19-patients supported with extracorporeal membrane oxygenation: an international study based on the COVID-19 critical care consortium
Source: J Intensive Care. 2024 May 6;12:18. doi: 10.1186/s40560-024-00726-2 (PMC11071263; doi:10.1186/s40560-024-00726-2)
Supplement: Supplementary file 1 — Additional file 1. Supplemental Material. [file 40560_2024_726_MOESM1_ESM.docx]

Hemorrhage and Thrombosis in COVID-19-Patients Supported with Extracorporeal Membrane Oxygenation: An International Study based on the COVID Critical Care Consortium

Supplemental Material

Maximilian **Feth**, MD^1^; Natasha **Weaver**, PhD^2,3^; Robert B. **Fanning**, BBiomed, MD^4,5^; Sung-Min **Cho**, DO, MHS^6,7^; Matthew J. **Griffee**, MD^8,9^; Mauro **Panigada**, MD^10^; Akram M. **Zaaqoq** MD, MPH^11,12^; Ahmed **Labib**, MBBCh(Hons), FRCA, FFICM^13^; Glenn JR **Whitman**, MD^6^; Rakesh C. **Arora**, MD, PhD, FACS, FRCSC^14,15^; Bo S. **Kim,** MD^6^; Nicole **White**, PhD^16^; Jacky Y. **Suen**, PhD^2,17^; Gianluigi **Li Bassi**, MD, PhD^2,16, 17,19,21^; Giles J. **Peek**, MD, FRCS, FFICM, FELSO^21^; Roberto **Lorusso**, MD, PhD^22^; Heidi **Dalton**, MD, MCCM, FELSO^23^; John F. **Fraser**, MBChB, PhD, FRCP(Glas), FRCA, FFARCSI, FCICM, FELSO^2,17,18,19^; Jonathon P. **Fanning**, MBBS, PhD, FRACP, FANZCA, FCICM^2,17,18, 24, 25^*; on behalf of* *the COVID-19 Critical Care Consortium*.

**Corresponding Author**

Jonathon P. Fanning

Critical Care Research Group

Level 3, Clinical Sciences Building, The Prince Charles Hospital

Chermside, 4032, Queensland, Australia

Email: [j.fanning@uq.edu.au](mailto:j.fanning@uq.edu.au)

eTable 1 List of recruiting sites and relevant IRB approval

| **Site Name** | **Ethics Committee Name** | **Ethics Approval Number** |
| --- | --- | --- |
| Adult ICU Saiful Anwar Hospital | Health Research Ethics RSUD Dr. Saiful Anwar Malang | 400/131/K.3/302/2020 |
| Al-Adan Hospital | Kuwait Ministry of Health Ethics Committee | 1403/2020 |
| Allegheny General Hospital | Allegheny-Singer Research Institute (ASRI)-WPAHS Institutional Review Board | 2020-113 |
| Avera McKennan Hospital & University Health Center | Avera Institutional Review Board | IRB00001096 |
| Barmherzige Bruder Regensburg | Ethics Committee at the University of Regensburg | N/A |
| Baylor All Saints Medical Centre | Baylor Scott & White Research IRB | 345512 |
| Baylor Scott | Baylor Scott & White Research IRB | 345512 |
| Baylor University Medical Centre | Baylor Scott & White Research IRB | 345512 |
| Beth Israel Deaconess Medical Center | Beth Israel Deaconess Medical Center Committee on Clinical Investigations | 2020P000355 |
| Box Hill Hospital | The Alfred Ethics Committee | Alfred 108/20 |
| Carilion Clinic | Carilion Clinic Institutional Review Board | IRB-20-863 |
| Chiba University Hospital | Research Ethics Committees of Graduate School of Medicine, Chiba University | 3704 |
| Chonnam National University Hospital | Chonnam National University Hospital Institutional Review Board | CNUH-2020-070 |
| Civil Hospital Marie Curie, Brussels | Ethics Committee CHU-Charleroi | P20/27_24/03 (B3252020) |
| Clinica Pasteur de Neuquen (University of Comahue) | Teaching and Research Committee of the Clinical Pasteur | N/A |
| Cleveland Clinic - Florida | Cleveland Clinic Institutional Review Board | IRB#20-507 |
| Cleveland Clinic Abu Dhabi | Cleveland Clinic Abu Dhabi Research Ethics Committee | A-2020-086 |
| Cleveland Clinic - Ohio | Cleveland Clinic Institutional Review Board | IRB#20-507 |
| Clinica Alemana De Santiago | Scientific Ethics Committee, University of Development Center for Bioethics, Faculty of Medicine, Clinica Alemana de Santiago | 2020-25 |
| Clinica Las Condes, Chile | Ethics Committee of Clinica Las Condes | 2020P000355 |
| Clinica Valle del Lili | Biomedical Research Ethics Committee of Fundacion Valle del Lilli | 121-2020 |
| Dr Sardjito Government Hospital - Paediatric | Medical and Health Research Ethics Committee (MHREC), Dr Sardjito General Hospital | KE/FK/0787/EC/2020 |
| Fatmawati Hospital | Human Resources and Education Jakarta | DM 01.01/VIII.2/266/2020 |
| Fondazione IRCCS Policlinico of Milan (Fondazione IRCCS Ca' Granda Ospedale Maggiore Policlinico) | Milan Area 2 Ethics Committee | 7785390 |
| Fondazione Policlinico Universitario Agostino Gemelli IRCCS, Rome, Italy | Fondazione Policlinico Gemelli Ethics Committee | 3029 |
| Foothills Hospital | Conjoint Health Research Ethics Board, University of Calgary | REB20-0455 |
| Fujieda Municipal General Hospital | Ethics Committee of Fujieda Municipal General Hospital | No. 3 |
| Fukuoka University | Not Provided | H20-05-001 |
| Fundación Cardiovascular de Colombia | Scientific Technical Committee of the Cardiovascular Foundation of Colombia | No.164 of 2020 |
| Galway University Hospital | National Research Ethics Committee for COVID-19-related Health Research (NREC COVID-19), Ireland | 20-NREC-COV-017 |
| Geelong Hospital, Barwon Health | The Alfred Ethics Committee | Alfred 108/20 |
| Gold Coast Hospital | The Alfred Ethics Committee | Alfred 108/20 |
| Groote Schuur Hospital | Faculty of Health Sciences, Human Research Ethics Committee, University of Cape Town | 205/2020 |
| Hamad General Hospital | Medical Research Center, Hamad Medical Corporation, Doha, Qatar | MRC-05-006 |
| Harapan Kita National Heart Centre Hospital | Health Research Ethics of National Cardiac Center Harapan Kita Hospital | LB.02.01/VII/419/KEP. 026/2020 |
| Hartford HealthCare | Hartford HealthCare Human Research Protection Program Institutional Review Board | HHC-2020-0068 |
| Hiroshima University | Hiroshima University Epidemiological Research Ethics Board. | E1963 and 2.4.4 |
| Hospital Alemán | Independent Ethics Committee of Hospital Alemán CEIHA | N/A |
| Hospital Clinic, Barcelona | Clinical Research Ethics Committee Hospital Clinic Barcelona | HCB/2020/0370 |
| Hospital de Clinicas, Argentina | Ethics Committee of the Hospital de Clinicas | N/A |
| Hospital du Sacre Coeur | Ethics Committee for Research and Development of new technologies of the ICM, Montreal Heart Institute | Project Number: MP-33-2020-2776 |
| Hospital Emergencia Ate Vitarte | Ministry of Health Peru - Hospital Emergencia Ate Vitarte | 009-2020-ADI-HEAV |
| Hospital Nuestra Señora de Gracia Zaragoza | Clinical Research Ethics Committee Hospital Clinic Barcelona | HCB/2020/0370 |
| Hospital Universitari Sagrat Cor | Clinical Research Ethics Committee Hospital Group Quironsalud-Catalunya | 2020/65-UCI-HUSC |
| Hospital Universitario Sant Joan d'Alacant | Clinical Research Ethics Committee Hospital Clinic Barcelona | HCB/2020/0370 |
| Hospital Vall D Hebron | Research Ethics Committee with Medicines and Research Projects Committee of Hospital University Vall D'Hebron | 417 |
| Hospitales Puerta de Hierro, Mexico | Research Ethics Committee of Hospital Puera de Hierro | 3/30/2020 |
| Hospital Mount Sinai Medical Center | Institutional Review Board of Mount Sinai Medical Center | FWA00000176 |
| Hospital Verge de la Cintade Tortosa | Ethical Medical Research Committee of the Pere Virgili Institute of Health Research | 123/2020 |
| Houston Methodist Hospital | Houston Methodist Research Institute Institutional Review Board | PRO00026837 |
| Hyogo Prefectural Kakogawa Medical Center | Ethics Committee of Hyogo Prefectural Kakogawa Medical Center | R2-7 |
| INCOR (Universidade de São Paulo) | National Commission on Research Ethics, Brazil | 70.719-040 |
| INOVA Fairfax Medical Center, USA | Inova Health System Institutional Review Board | U20-03-3980 |
| Instituto Nacional Del Tórax | Eastern Metropolitan Health Service Scientific Ethics Committee. | N/A |
| ISMETT | IRCCS ISMETT sectional Ethics Committee | IRRB/09/20 |
| Johns Hopkins University | John Hopkins Medicine Institutional Review Board | IRB00247449 |
| Keimyung University Dong San Hospital | Keimyung University Dong San Medical Center | DSMC202004015-HE004 |
| Kimitsu Chuo Hospital | Not Provided | 526 |
| King Faisal Specialist Hospital and Research Center | Research Ethics Committee King Faisal Specialist Hospital and Research Centre | C380/800/41 |
| Klinik für Innere Medizin II | Ethics Committee University of Regensburg | 20-1747-101 |
| Klinikum Passau | Ethics Committee at the University of Regensburg | N/A |
| Kouritu Tousei Hospital | Ethics Committee of Kouritu Tousei Hospital | 885 |
| Kuwait ECLS program, Al-Amiri & Jaber Al-Ahmed Hospitals | Kuwait Ministry of Health Ethics Committee | 1405/2020 |
| Kyoto Medical Centre | Ethics Committee of Kyoto Medical Centre | 20-001 |
| Kyoto Prefectural University of Medicine | Ethics Committee of Kyoto Prefectural University of Medicine | ERB-C-1716 |
| Kyung Pook National University Chilgok Hospital | Kyung Pook National University Chilgok Hospital Institutional Review Board | KNUCH 2020-04-060 |
| Lancaster General Health | Lancaster General Hospital Institutional Review Board | 2020-24 |
| Lankenau Institute of Medical Research | Main Line Hospitals Institutional Review Board (MLH IRB) | E-20-5039 |
| Launceston Hospital | Tasmania Health and Medical Human Research Ethics Committee | 21666 |
| Legacy Emanuel Medical Center | Legacy Health Institutional Review Board | 1820 |
| London Health Sciences Centre | Western University Health Science Research Ethics Board | Project ID: 115907 |
| Maastricht University Medical Centre | Medical Ethics Review Committee, Maastricht University | METC2020-1566 |
| Manipal Hospital Whitefield | Ethics Committee of Manipal Hospitals, Bangalore | N/A |
| Mar del Plata Medical Foundation Private Community Hospital | Institutional Council of the Review of Research Studies (CIREI), Private Hospital of Community or Mar del Plata | 2919/2143/2020 |
| Mater Misericordiae University Hospital, Ireland | Mater Misericodiae University Hospital | 1/378/2137 |
| Mayo Clinic College of Medicine - Arizona | Mayo Clinic Institutional Review Board | 20-004851 |
| McGill University Health Centre | McGill University Health Centre Research Ethics Board | Project Number: 2020-6571 |
| Medical College of Wisconsin, USA | Medical College of Wisconsin Froedtert Hospital Institutional Review Board | PRO00037778 |
| Medical University of Vienna | Ethics Committee at Medical University of Vienna | EC Nr: 1276/2020 |
| Medizinische Klinik und Poliklinik II, Munich | Ethics Committee at the University of Regensburg | N/A |
| MedStar Washington Hospital Centre | Medstar Health Research Institute Institutional Review Board | STUDY00002284 |
| Nagoya University Hospital | Not Provided | 2020-0073-2 |
| National Taiwan University Hospital | Research Ethics Committee National Taiwan University Hospital | 202002009RINA |
| North Estonia Medical Centre, Tallin | Ethics Committee for Human Research of the Institute for Health Development (TAEIK) | Application Number 2121 |
| Northwell Health | Feinstein Institutes for Medical Research Northwell Health Institutional Review Board. | 25-0591 |
| Obihiro-Kosei General Hospital | Ethics Committee of Obihiro-Kosei General Hospital | 2020-069 |
| Ochsner Clinic Foundation | Ochsner Clinic Foundation Institutional Review Board | FWA00002050 |
| Ohio State University | The Ohio State Biomedical Sciences Institutional Review Board | 2020H0139 |
| Oklahoma Heart Institute | Hillscrest medical Center Institutional Review Board | N/A |
| Oregon Health and Science University Hospital | Oregon Health and Science University Institutional Review Board | STUDY00021357 |
| Ospedale Molinette Torino | Inter-Company Ethics Committee A.O.U City of Health and Science of Turin- A.O Maurizian Order of Turin- A.S.L City of Turin | 00103/2020 |
| Ospedale San Gerardo | Brianza Ethics Committee | N/A |
| Ospedale San Paolo | Milan Area 1 Ethics Committee | P/08/05/2020 |
| Pamela Youde Nethersole Eastern Hospital | Hong Kong East Cluster Research Ethics Committee (HKEC REC) | HKECREC-2020-0.16 |
| Penn Medicine | University of Pennsylvania Institutional Review Board | 842836 |
| Persahabatan Hospital | Ethic Committee of Health Research of Persahabatan Hospital | 61/KEPK-RSUPP/6/2020 |
| PICU Saiful Anwar Hospital | Health Research Ethics RSUD Dr. Saiful Anwar Malang | 400/131/K.3/302/2020 |
| Piedmont Atlanta Hospital, USA | Piedmont Healthcare Institutional Review Board | 1591828-1 |
| Policlinico di S. Orsola, Università di Bologna, Italy | Ethics Committee of the Vasta Emilia-Romagna Region | 1321/2020 |
| Policlinico of Padova, Padova | Ethics Committee for Clinical rials of the Province of Padua | 4851/A0/20 |
| Presbyterian Hospital Services | Presbyterian Healthcare Services Institutional Review Board | 1602073-1 |
| Princess Margaret Hospital, Hong Kong | Kowloon West Cluster Research Ethics Committee (KWC-REC) | KW/EX-20-051(154-10) |
| Prof Dr R. D. Kandou Central Hospital - Adult | Health Research Ethics Commission (KEPK) RSUP Prof Dr. RD Kandou | PP 04.03/XIX 2/126 12020 |
| Providence Saint John's Health Centre | Providence St Johns Health Institutional Review Board | STUDY2020000272 |
| Queen Elizabeth Hospital, Hong Kong | Research Ethics Committee (Kowloon Central/Kowloon East) | KC/KE-20-0064/ER-3 |
| Queen Mary Hospital the University of Hong Kong | University of Hong Kong/Hospital Authority Hong Kong West Cluster (HKU/HA HKW IRB) | UW 20-143 |
| Rinku General Medical Center | Ethics Committee of Rinku General Medical Center | 2020-006 |
| Rio Hortega University Hospital | Ethics Committee for the Clinical Research of the West-Valladolid Sanitary Area | PI077/20 |
| Rochester General Hospital | Rochester Regional Health Institutional Review Board | IRB 2018 B Hall |
| Royal Children's Hospital | The Alfred Ethics Committee | Alfred 108/20 |
| Royal North Shore Hospital | The Alfred Ethics Committee | Alfred 108/20 |
| Royal Prince Alfred Hospital | The Alfred Ethics Committee | Alfred 108/20 |
| RSPI Prof Dr Sulianti Saroso | Infectious Disease Hospital Prof. Dr Sulianti Saroso Committee of Health Research Ethics. | 09/XXXVIII.10/XI/2020 |
| RSUD Dr. Soetomo | Committee of Health Research Ethics RSUD Dr Soetomo Surabaya | 0096/KEPK/XI/2020 |
| Rush University | Rush University Medical Center Institutional Review Board | 20040706-IRB01 |
| Saiseikai Utsunomiya Hospital | Utsunomiya Hospital, Tochigi Prefecture | 2019 71 |
| Saiseikai Senri Hospital | Not provided | 20408 |
| San Martino Hospital | Reginal Ethics Committee of Liguria | 163/2020 |
| Sao Camilo Cura D'ars | Comitē de Ētica, Hospital Distrital Gonzaga Mota Messejana | N/A |
| Sāo Joāo Hospital Centre, Portugal | Ethics Committee for Health of the University Hospital Center of Sāo Joāo/Faculty of Medicine of the University of Porto | ID Number: 11002511 |
| Seoul National University Bundang Hospital | Seoul National University Bundang Hospital Institutional Review Board | B-2003/601-305 |
| Severance Hospital, Seoul | Yonsei University Health System, Severance Hospital, Institutional Review Board | 4-2020-0099 |
| Sinai Health Systems | University Health Network Research Ethics Board | Project ID: 2129 |
| Siriraj Hospital | Siriraj Institutional Review Board | 230/2563(IRB3) |
| Sozialmedizinisches Zentrum Süd - Kaiser-Franz-Josef-Spital | Ethics Committee of the City of Vienna | EK 20-064-VK |
| St Christopher's Hospital for Children | Drexel University Institutional Review Board | 2005007860 |
| St George Hospital | The Alfred Ethics Committee | Alfred 108/20 |
| St Vincent's Hospital Sydney | The Alfred Ethics Committee | Alfred 108/20 |
| St. Boniface Hospital, Manitoba | University of Manitoba Health Research Board | HS23821 |
| Tartu University Hospital, Tartu | Ethics Committee for Human Research of the Institute for Health Development (TAEIK) | Application Number 2121 |
| Teine Keijinkai Hospital | The Institutional Review Board at Teine Keijinkai Hospital | 2-019180-00 |
| The Alfred Hospital | The Alfred Ethics Committee | Alfred 108/20 |
| The Heart Hospital Baylor Plano | Baylor Scott & White Research IRB | 345512 |
| The University of Utah | Institutional Review Board the University of Utah | IRB_00131842 |
| Tohoku Medical and Pharmaceutical University | Not Provided | 2020-2-011 |
| Tokyo Metropolitan Tama Medical Center | Ethics Committee of Tokyo Metropolitan Tama Medical Center | 72 |
| Tufts Medical Centre | Tufts Health Sciences Institutional Review Board | MOD-01-STUDY00000808 |
| UH Cleveland Hospital | University Hospitals Institutional Review Board | STUDY20200428 |
| Uniklinik (University Hospital Frankfurt) | Ethics Committee of the Faculty of Medicine at Goethe University | 20-668 |
| University Airlangga Hospital (Paediatric) | Airlangga University Hospital Research Committee. | 178-A/KEP/2020 |
| University Hospital in Krakow | Bioethical Commission, The University Jagiellonian | 1072.6120.86.2020 |
| University of California San Francisco - Fresno | Community Medical Centers Institutional Review Board | 2020021 |
| University of Chicago | The University of Chicago Biological Sciences Division Institutional Review Board | IRB20-0579 |
| University of Cincinnati | University of Cincinnati Institutional Review Board | 2020-0406 |
| University of Iowa | University of Iowa Institutional Review Board | 202004380 |
| University of Nebraska Medical Center | University of Nebraska Medical Centre Institutional Review Board | 233-20-EP |
| University of Alabama at Birmingham Hospital | University of Alabama Institutional Review Board | IRB-300005234 |
| University of Florida | University of Florida Institutional Review Board | IRB202000933 |
| University of Oklahoma Health Sciences Center | University of Oklahoma Institutional Review Board for the Protection of Human Subjects | 11978 |
| UT Southwestern | University of Texas Southwestern Medical Center Institutional Review Board | N/A |
| Washington University in St. Louis, USA | The Washington University in St. Louis Institutional Review Board | 202004038 |
| Yokohama City University Medical Center | Ethics Committee of Yokohama City University Medical Center | B200500022 |
| Hospital Clinic De Barcelona | These sites received approvals to participate in the CIBERESUCICOVID study. CIBERSUCICOVID and COVID Critical executed a data sharing agreement to merge data into COVID Critical. Hospital Clinic Barcelona is considered the data controller for CIBERESUCICOVID so therefore ethics was approved by Clinical Research Ethics Committee, Hospital Clinic of Barcelona. | Ethics approval number HCB/2020/0370 |
| Hospital Universitario Sant Joan DAlacant |  |  |
| HULA |  |  |
| Hospital Nuestra Senora De Gracia |  |  |
| Hospital De Bellvitge |  |  |
| Clinica Sagrada Familia |  |  |
| Hospital Vall D’Hebron |  |  |
| Hospital Clinico Universitario De Valladolid |  |  |
| Hospital Universitario De Leon |  |  |
| Hospital Arnau De Vilanova De Lleida |  |  |
| Hospital San Pedro De Alcantara |  |  |
| Hospital Sagrat Cor |  |  |
| HUIL |  |  |
| Hospital La Fe De Valencia |  |  |
| Hospital Universitari Mutua Terrassa |  |  |
| Hospital Del Mar |  |  |
| Hospital Universitario Central De Asturias |  |  |
| Hospital De Mataro |  |  |
| Hospital Universitario De Valme |  |  |
| CHUO |  |  |
| Caupa |  |  |
| Hospital La Paz |  |  |
| Hospital Alvaro Cunqueiro |  |  |
| Hospital Universitario De Salamanca |  |  |
| Tortosa |  |  |
| Hospital Clinic Universitari De Valencia |  |  |
| Husll |  |  |
| Hospital Santa Maria De Lleida |  |  |
| Hospital Jerez De La Frontera |  |  |
| Hospital Universitario San Agustin |  |  |
| Parc Tauli |  |  |
| Hospital Clinico Universitario De Santiago |  |  |
| HUMV |  |  |
| Hospital Germans Trias I Pujol |  |  |
| Hospital De Torrejon |  |  |
| H.U.Basurto |  |  |
| Hospital Universitari Joan XXIII De Tarragona |  |  |
| Hospital Universitario De Cruces |  |  |
| Hospital De La Princesa |  |  |
| Hospital Universitario Reina Sofia (Hurs) |  |  |
| Hugc Dr Negrin |  |  |
| Hospital Son Espases |  |  |
| Hospital Universitario Hm Sanchinarro |  |  |
| Hospital Universitario Virgen Del Rocio |  |  |
| Hospital Virgen Macarena |  |  |
| Hospital San Juan De Dios Bormujos Aljarafe |  |  |
| Hospital General Universitario Gregorio Marano |  |  |
| Hospital De Getafe |  |  |
| Hospital Ramon Y Cajal |  |  |
| Hospital Universitario Rio Hortega |  |  |
| Hospital Universitario Principe De Asturias |  |  |
| HPE |  |  |
| Hospital 12 De Octubre |  |  |
| Hospital De Mostoles |  |  |
| Policlinico di Milano | On behalf of these sites, as data controller for the Italian sites, Policlinico of Milan executed a data sharing agreement with the COVID Critical study. Ethics was approved by the Milan Area 2 Ethics Committee. | Ethics approval number 7785390 |
| Niguarda |  |  |
| San Gerardo |  |  |
| Mantova Poma |  |  |
| Varese Sette Laghi |  |  |
| Humanitas Research Hospital |  |  |
| San Giovanni Molinette |  |  |
| Sant'Orsola Bologna |  |  |
| ASST Lecco - Ospedale di Merate |  |  |
| ASST Lecco - Ospedale A. Manzoni |  |  |
| ASST Nord Milano - Ospedale Edoardo Bassini - Cinisello Balsamo |  |  |
| ASST-MONZA, Ospedale di Desio |  |  |
| AULSS 5 Polesana - Ospedale di Rovigo e Ospedale di Trecenta |  |  |
| AULSS 9 Scaligera - Ospedale Magalini di Villafranca |  |  |
| Azienda Ospedaliera di Perugia |  |  |
| Azienda Sanitaria Universitaria Friuli Centrale - Udine |  |  |
| Azienda Ospedaliero - Universitaria di Modena |  |  |
| Policlinico Universitario Fondazione Agostino Gemelli - Roma |  |  |
| Azienda Ospedaliera Mater Domini - Catanzaro |  |  |
| Azienda Ospedaliera Universitaria Federico II |  |  |
| Azienda Ospedaliero-universitaria di Ferrara |  |  |
| Azienda Ospedaliera Universitaria Foggia - Ospedali Riuniti |  |  |
| AOU - Ospedali Riuniti di Ancona |  |  |
| Policlinico Universitario Paolo Giaccone - Palermo |  |  |

eTable 2 Data completeness. SOFA, sequential organ failure assessment, APACHE, acute physiology and chronic health evaluation, ECMO, extracorporeal membrane oxygenation

| **Characteristic,**  **n (%) missing** | **Neither**  **(n=779)** | **Combined (n=52)** | **Hemorrhagic only (n=252)** | **Thrombotic only (n=165)** | **TOTAL** |
| --- | --- | --- | --- | --- | --- |
| Age (years) | 0 | 0 | 0 | 0 | 0 |
| Body mass index (kg/m2) | 90 (12%) | 4 (7.7%) | 30 (12%) | 22 (13%) | 146 (12%) |
| SOFA score | 594 (76%) | 34 (65%) | 134 (53%) | 127 (77%) | 889 (71%) |
| APACHE II score | 608 (78%) | 37 (71%) | 140 (56%) | 137 (83%) | 922 (74%) |
| Time from admission to mechanical ventilation (days) | 402 (52%) | 21 (40%) | 132 (52%) | 72 (44%) | 627 (50%) |
| Time from admission to ECMO (days) | 114 (15%) | 1 (1.9%) | 28 (11%) | 11 (6.7%) | 154 (12%) |
| Mechanical ventilation (days) | 77 (10%) | 1 (1.9%) | 12 (4.8%) | 11 (6.7%) | 101 (8.1%) |
| ECMO (days) | 98 (13%) | 0 (0%) | 12 (4.8%) | 4 (2.4%) | 114 (9.1%) |
| Hospital length of stay (days) | 97 (12%) | 5 (10%) | 30 (12%) | 15 (9.1%) | 147 (12%) |
| ICU length of stay (days) | 82 (11%) | 4 (7.7%) | 21 (8.3%) | 16 (10%) | 123 (10%) |
| Time from ICU admission to death (days) | 57/304 (19%) | 0/24 | 3/141 (2.2%) | 10/58 (17%) | 70/462 (15%) |
| Time from ICU admission to discharge alive (days) | 0/393 | 0/24 | 0/90 | 0/91 | 0/570 |
| Time from admission to death (days) | 54/304 (22%) | 0/24 | 1/141 (0.7%) | 10/58 (17%) | 65/462 (14%) |
| Time from admission to discharge alive (days) | 19/393 (4.8%) | 0/24 | 8/90 (8.9%) | 1/91 (1.1%) | 28/570 (4.9%) |
| Discharge disposition | 0 | 0 | 0 | 0 | 0 |
| Mortality at 28 days | 14 (1.8%) | 0 (0%) | 7 (2.8%) | 3 (1.8%) | 24 (1.9%) |
| Mortality at 90 days | 14 (1.8%) | 0 (0%) | 7 (2.8%) | 3 (1.8%) | 24 (1.9%) |
| Ethnicity | 185 (24%) | 4 (7.7%) | 15 (6%) | 32 (19%) | 236 (19%) |
| comorbidity_obesity | 173 (22%) | 1 (1.9%) | 9 (3.6%) | 5 (3%) | 188 (15%) |
| Chronic cardiac disease | 141 (18%) | 1 (1.9%) | 8 (3.2%) | 7 (4.2%) | 157 (13%) |
| Chronic kidney disease | 142 (18%) | 1 (1.9%) | 9 (3.6%) | 8 (4.8%) | 160 (13%) |
| Chronic neurological disorder | 177 (23%) | 1 (1.9%) | 10 (4%) | 7 (4.2%) | 195 (16%) |
| Chronic haematologic disorder | 178 (23%) | 1 (1.9%) | 9 (3.6%) | 9 (5.5%) | 197 (16%) |
| Diabetes | 179 (23%) | 2 (3.8%) | 15 (6%) | 16 (10%) | 212 (17%) |
| Hypertension | 143 (18%) | 1 (1.9%) | 5 (2%) | 6 (3.6%) | 155 (12%) |
| Smoking | 167 (21%) | 0 (0%) | 11 (4.4%) | 10 (6.1%) | 188 (15%) |
| Malignant neoplasm | 178 (23%) | 1 (1.9%) | 11 (4.4%) | 8 (4.8%) | 198 (16%) |
| Prone positioning (mechanical ventilation) | 280 (36%) | 0 (0%) | 13 (5.2%) | 28 (17%) | 321 (26%) |
| Prone positioning (before ECMO) | 376 (48%) | 2 (3.8%) | 39 (15%) | 49 (30%) | 466 (37%) |
| Inhaled nitric oxide | 284 (36%) | 1 (1.9%) | 10 (4%) | 25 (15%) | 320 (26%) |
| Vasopressor use | 208 (27%) | 0 (0%) | 8 (3.2%) | 4 (2.4%) | 220 (18%) |
| Neuromuscular blockade (before ECMO) | 358 (46%) | 3 (5.8%) | 43 (17%) | 49 (30%) | 453 (36%) |
| Tracheostomy | 198 (25%) | 1 (1.9%) | 11 (4.4%) | 2 (1.2%) | 212 (17%) |
| Anticoagulation therapy | 474 (61%) | 16 (31%) | 130 (52%) | 60 (36%) | 680 (54%) |

eTable 3 ECMO Characteristics

| **Characteristic** | **Class or Statistic** | **Neither**  **(n=779)** | **Both  (n=52)** | **Hemorrhagic only (n=252)** | **Thrombotic only (n=165)** |
| --- | --- | --- | --- | --- | --- |
| ECMO type | Venous-Arterial | 28 (5.9%) | 9 (18%) | 10 (4.7%) | 10 (6.7%) |
|  | Venous-Venous | 464 (94%) | 41 (82%) | 204 (95%) | 139 (93%) |
| Drainage cannula site | Left femoral vein | 113 (28%) | 13 (27%) | 56 (27%) | 29 (24%) |
|  | Left internal jugular vein | 5 (1.2%) | 1 (2.1%) | 2 (1.0%) | 1 (0.8%) |
|  | Right femoral vein | 171 (43%) | 18 (38%) | 106 (51%) | 67 (55%) |
|  | Right internal jugular vein | 112 (28%) | 16 (33%) | 45 (22%) | 25 (20%) |
| Location of cannulation | Bedside | 4 (1.0%) |  | 1 (0.5%) | 1 (0.8%) |
|  | Another hospital, following which the patient was transferred to participating hospital | 125 (30%) | 13 (26%) | 77 (36%) | 30 (24%) |
|  | Same hospital | 286 (69%) | 37 (74%) | 137 (64%) | 95 (75%) |
| Return cannula insertion site | Left femoral artery | 10 (2.6%) | 2 (4.4%) | 7 (3.4%) | 5 (4.2%) |
|  | Left femoral vein | 28 (7.2%) | 2 (4.4%) | 20 (9.8%) | 10 (8.3%) |
|  | Left internal jugular vein | 27 (6.9%) | 1 (2.2%) | 14 (6.9%) | 7 (5.8%) |
|  | Right femoral artery | 21 (5.4%) | 1 (2.2%) | 4 (2.0%) | 2 (1.7%) |
|  | Right femoral vein | 108 (28%) | 18 (40%) | 59 (29%) | 26 (22%) |
|  | Right internal jugular vein | 196 (50%) | 21 (47%) | 100 (49%) | 70 (58%) |
| Maximum daily blood flow rate (L/min) | Mean (SD) | 4.49 (3.85, 5.00) | 4.46 (4.00, 4.72) | 4.50 (4.00, 5.16) | 4.25 (3.80, 4.76) |
| Mean daily blood flow rate (L/min) | Mean (SD) | 4.17 (3.55, 4.70) | 3.92 (3.51, 4.45) | 4.10 (3.65, 4.64) | 3.98 (3.50, 4.36) |
| Mean daily blood flow rate/kg bodyweight (mL/kg bodyweight/ day) | Mean (SD) | 44.35 (36.01, 53.61) | 44.66 (36.41, 51.40) | 45.86 (39.36, 53.75) | 42.58 (36.48, 50.00) |
| Any circuit change(s) | Yes | 16% | 26% | 22% | 12% |
| Number of circuit changes* | Mean (SD) | 1.51 (1.16) | 2.08 (2.02) | 1.30 (0.61) | 1.31 (0.60) |

The term “circuit” refers to technical components needed to provide ECMO, e.g. the membrane lung, tubing, cannulas and a centrifugal pump. One or several of these components potentially need to be changed in order to prevent or solve complications such as failure of the membrane lung or thrombosis of the pump (circuit change).

## **Appendix 1: Covid Critical Care Consortium Steering Committee and Key Personnel**

**S****teering Committee**

Gianluigi Li Bassi MD^1, 3, 4,5, 7, 8^, PhD; Jacky Y. Suen BSc^1, 2^, PhD; Heidi J. Dalton MD, MCCM^9^; John Laffey, MA, MD^10^; Daniel Brodie, MD^11^; Eddy Fan, MD, PhD^12^; Antoni Torres, MD, PhD, FERS ATS Fellow^4, 13 36 37^; Davide Chiumello, MD^14^; Alyaa Elhazmi^15^; Carol Hodgson, PT, PhD^16,31^; Shingo Ichiba, MD^17^; Carlos Luna, MD^18^; Srinivas Murthy, MD^19^; Alistair Nichol, MD, PhD^16, 21,31^; Pauline Yeung Ng, MD^22^; Mark Ogino, MD^23^;  Eva Marwali, MD, PhD^35^; Giacomo Grasselli MD^33, 34^, PhD; Robert Bartlett, MD^25^; Aidan Burrell, MBBS, PhD^26, 27^ ￼; Muhammed Elhadi MBBCh^38^; Anna Motos ^39,40^; Ferran Barbé MD, PhD^41,42^; Alberto Zanella MD^33^; and John F. Fraser MBChB, PhD, FRCP(Glas), FFARCSI, FRCA, FCICM, FELSO^1, 3, 5, 7, 8, 43^ **on behalf of the COVID-19 Critical Care Consortium**

**Affiliations**

1. Critical Care Research Group, The Prince Charles Hospital, Brisbane, Australia
2. Faculty of Medicine, The University of Queensland, Brisbane, Australia
3. University of Queensland, Brisbane, Australia
4. Institut d'Investigacions Biomèdiques August Pi i Sunyer (IDIBAPS), Barcelona, Spain
5. Queensland University of Technology, Brisbane, Australia
6. School of Public Health, Queensland University of Technology, Brisbane, Australia
7. St Andrew’s War Memorial Hospital, UnitingCare Hospitals, Brisbane Australia
8. Wesley Medical Research, Brisbane, Australia
9. INOVA Fairfax Medical Center, Heart and Vascular Institute, Falls Church VA, USA
10. Anaesthesia and Intensive Care Medicine, Galway University Hospitals, and School of Medicine, National University of Ireland, Galway, Ireland
11. Department of Medicine, Columbia University College of Physicians and Surgeons, New York-Presbyterian Hospital, NY, NY, USA
12. Interdepartmental Division of Critical Care Medicine, University of Toronto, Toronto, Canada
13. Servei de Pneumologia. Hospital Clinic de Barcelona, Barcelona, Spain
14. Ospedale San Paolo, Milan, Italy
15. Dr. Sulaiman Alhabib Medical Group - Research Center, Riyadh, Saudi Arabia
16. Australian and New Zealand Intensive Care Research Centre, Department of Epidemiology and Preventive Medicine, School of Public Health, Monash University, Melbourne, Australia
17. Department of Clinical Engineering / Department of Intensive Care Medicine, Tokyo Women’s Medical University Hospital, Japan
18. División Neumonología, Hospital de Clínicas, UBA, Buenos Aires, Argentina
19. Department of Pediatrics, Faculty of Medicine, University of British Columbia, Vancouver, Canada
20. Australian and New Zealand Intensive Care Research Centre, Department of Epidemiology and Preventive Medicine, School of Public Health, Monash University, Melbourne, Australia
21. University College Dublin-Clinical Research Centre at St Vincent’s University Hospital, Dublin
22. Division of Respiratory and Critical Care Medicine, The University of Hong Kong, Hong Kong, China
23. Nemours Alfred I duPont Hospital for Children, Wilmington, DE, USA
24. Fondazione IRCCS Ca' Granda Ospedale Maggiore Policlinico, Department of Pathophysiology and Transplantation, University of Milan, Milan, Italy
25. University of Michigan Medical Center, Ann Arbor, Michigan, USA
26. Australian and New Zealand Intensive Care Research Centre (ANZIC-RC), School of Public Health and Preventive Medicine, Monash University, Melbourne, Victoria, Australia.
27. Department of Intensive Care and Hyperbaric Medicine, The Alfred Hospital, Melbourne, VIC, Australia.
28. Australian Centre for Health Services Innovation (AusHSI) and Centre for Healthcare Transformation, School of Public Health & Social Work, Queensland University of Technology (QUT), Brisbane, Queensland, Australia
29. Child Health Research Centre, Faculty of Medicine, The University of Queensland, Brisbane, Queensland, Australia
30. ISARIC, Centre for Tropical Medicine and Global Health, University of Oxford, Oxford, UK
31. Department of Physiotherapy, Alfred Hospital, Melbourne, Australia
32. Department of Intensive Care, Alfred Hospital, Melbourne, Australia
33. Department of Anesthesia, Intensive Care and Emergency, Fondazione IRCCS Ca’ Granda Ospedale Maggiore Policlinico, Milan, Italy
34. Department of Pathophysiology and Transplantation, University of Milan, Milan, Italy
35. National Cardiovascular Center Harapan Kita, Jakarta, Indonesia
36. Catalan Institution for Research and Advanced Studies (ICREA), Barcelona, Spain
37. Centro de Investigación Biomédica en Red Enfermedades Respiratorias (CIBERES), Madrid, Spain
38. Faculty of Medicine, University of Tripoli, Tripoli, Libya
39. Centro de Investigación Biomedica En Red - Enfermedades Respiratorias (CIBERES), Barcelona, Spain.
40. Institut d'Investigacions August Pi i Sunyer (IDIBAPS), Barcelona, Universitat de Barcelona, Barcelona, Spain.
41. Translational Research in Respiratory Medicine, Respiratory Dept, Hospital Universitari Aranu de Vilanova and Santa Maria; IRBLleida, Lleida, Spain.
42. Centro de Investigación Biomedica En Red - Enfermedades Respiratorias (CIBERES), Barcelona, Spain
43. School of Medicine, Griffith University, Brisbane, Australia
44. **Appendix**

**Contributors**

| **Prefix/First Name/Last Name** | **Site Name** |
| --- | --- |
| Tala Al-Dabbous Huda Alfoudri Mohammed Shamsah | Al Adan Hospital |
| Khadeejeh Alfroukh  Zinah Aqeel Abdulzahra Bairmani  Khalid Jehad Khalid  Salsabeel M.A. Abukhalaf | Al Ahli Hospital |
| Mohammed Maher Hadhoud  Mohamed Fouad Abdrabo | Al Menshawy General Hospital |
| Mohamed Fathi | Al-Hawari Center of Surgical Speciality |
| Hasan Alhouri | Al-Mouwasat University Hospital - Damascus |
| Dr Hamza Shahla | Al-Thawra Teaching Hospital in Albayda |
| Qamrah Alhadad Matly Hanan | Aljufrah Clinical Isolation Center |
| Subbarao Elapavaluru Ashley Berg Christina Horn | Allegheny General Hospital |
| Ahmed Reda Mohamed Elsayed Abdelhalim  Amro Essam Amer  Cinderella Omar Rageh Elnaggar  Ahmed Ayman Hassan  Ali Abdelaziz  Mohamed Abdelhalim  Yehia Samir Shaaban Aly Orabi | Alexandria Main University Hospital |
| Zinah A. Alaraji  Mo'nes R. Muhaisen  Lana Almasri  Dana Mustafa  Shaher Hamdan  Yousef Al-Saba'a  Zaina Dalloul  Mohammed Alkahlout  Hamza Jaber  Osama Aldabbourosama  Alaa Abdalfattah Abdalhadi | Alshifa Hospital, Gaza |
| Aliae AR Mohamed Hussein  Zarief Kamel Emad  Sarah Khaled  Nouralsabah Mohamed  Ebtisam Hassanin  Abdelhafeez Hamdi  May Gamal  Ahmed Emad  Abdelrahman Ragab  Mohammed G Azizeldin | Assiut University Hospital |
| Almthani Hamza  Alsarrah Ali Mohammed Omer  Asgad Osman Abdalla Fadl alla  Asia Atif Abdelrahman Abdallahrs  Aml Ahmed Eltayeb  Maali khalid mohamed abdalla Alhasan  Esraa Hassan Abdelgaum  Aya Mustafa Ahmed  Lamees Adil Abdulbaqi  Omer Abdullah Mohammedelhassan  Musaab Mohammed Mohammed Ahmed  Maha TagElser Mohammed Ali | Atbara Teaching Hospital |
| Yunis Mayasi | Avera McKennan Hospital & University Health Centre |
| Stephan Schroll | Barmherzige Bruder Regansburg |
| Dan Meyer Jorge Velazco Ludmyla Ploskanych Wanda Fikes Rohini Bagewadi Marvin Dao Haley White  Alondra Berrios Laviena  Ashley Ehlers Maysoon Shalabi-McGuire Trent Witt | Baylor Scott & White Health |
| Lorenzo Grazioli Luca Lorini | Bergamo Hospital |
| E. Wilson Grandin Jose Nunez Tiago Reyes | Beth Israel Deaconess Medical Centre |
| Diarmuid O’Briain Stephanie Hunter | Box Hill Hospital |
| Mahesh Ramanan Julia Affleck | Caboolture Hospital |
| Hemanth Hurkadli Veerendra  Sumeet Rai Josie Russell-Brown Mary Nourse | Canberra Hospital |
| Mark Joseph Brook Mitchell Martha Tenzer | Carilion Clinic |
| Ryuzo Abe | Chiba University Graduate School of Medicine |
| Hwa Jin Cho In Seok Jeong | Chonnam National University Hospital |
| Nadeem Rahman Vivek Kakar  Ahmed Tamimi  Diala Zabalawi  Mohamed Elhennawi  Praveen Ghisulal  Sadaf Malik | Cleveland Clinic- Abu Dhabi |
| Nicolas Brozzi | Cleveland Clinic - Florida |
| Omar Mehkri Sudhir Krishnan Abhijit Duggal Stuart Houltham | Cleveland Clinic - Ohio |
| Jerónimo Graf | Clinica Alemana De Santiago |
| Roderigo Diaz Roderigo Orrego  Camila Delgado Joyce González Maria Soledad Sanchez Michael Piagnerelli Josefa Valenzuela Sarrazin | Clinica Las Condez |
| A/Prof. Gustavo Zabert Lucio Espinosa Paulo Delgado Victoria Delgado | Clinica Pasteur National- University of Comahue |
| Diego Fernando Bautista Rincón Angela Maria Marulanda Yanten Melissa Bustamante Duque | Clinica Valle de Lilli |
| Daniel Brodie | Medical ICU, Columbia College of Physicians and Surgeons, New-York-Presbyterian Hospital, NY, NY, USA |
| Khaled Abouelmagd | Dr. Mohammad Alfagih Hospital |
| Alyaa Elhazmi Abdullah Al-Hudaib | Dr Sulaiman Alhabib Medical Group – Research Center, Riyadh, Saudi Arabia |
| Jeff Javidfar  Maria Callahan  Andy Dong  Charles Crepy D'Orleans | Emory University Healthcare System |
| M. Azhari Taufik  Elizabeth Yasmin Wardoyo Margaretha Gunawan Nurindah S Trisnaningrum Vera Irawany Muhammad Rayhan | Fatmawati Hospital |
| Mauro Panigada Antonio Pesenti Alberto Zanella Giacomo Grasselli Sebastiano Colombo  Chiara Martinet  Gaetano Florio | Fondazione IRCCS Policlinico of Milan (Fondazione IRCCS Ca' Granda Ospedale Maggiore Policlinico) |
| Massimo Antonelli Simone Carelli Domenico L. Grieco | Fondazione Policlinico Universitario Agostino Gemelli IRCCS |
| Motohiro Asaki | Fujieda Municipal General Hospital |
| Kota Hoshino | Fukuoka University |
| Leonardo Salazar  Mary Alejandra Mendoza Monsalve | Fundación Cardiovascular de Colombia |
| John Laffey Bairbre McNicholas David Cosgrave  Minha Atif  Fadi Qutishat  Caoimhe Laffey  Michaeal Van Der Walt | Galway University Hospitals |
| Joseph McCaffrey Allison Bone  Jemma Trickey  Michelle Horton  Michelle Horton  Stephanie Pearce  Tania Salerno | Geelong Hospital |
| Akram Mohamed  Salem Alhaddad  Baliad Bakeer  Shames Haitam  Laila Shalabi  Mohammed Abodina Ahmed | Gheryan Central Hospital |
| Yusuff Hakeem | Glenfield Hospital |
| James Winearls Mandy Tallott | Gold Coast University Hospital |
| David Thomson  Ivan Joubert Christel Arnold-Day  Jenna Piercy  Richard van Zyl Smit Malcom Miller Lisa Seymour  Francois van Heyningen  Gilbert Teyangesikayi  David Fredericks | Groote Schuur Hospital |
| Ali Ait Hssain Jeffrey Aliudin Al-Reem Alqahtani Khoulod Mohamed Ahmed Mohamed Darwin Tan Joy Villanueva Ahmed Zaqout  Ahmed Labib | Hamad General Hospital - Weill Cornell Medical College in Qatar |
| Ethan Kurtzman Arben Ademi Ana Dobrita Khadija El Aoudi Juliet Segura | Hartford HealthCare |
| Gezy Giwangkancana | Hasan Sadikin Hospital (Adult) |
| Shinichiro Ohshimo | Hiroshima University |
| Javier Osatnik | Hospital Alemán |
| Anne Joosten | Hospital Civil Marie Curie |
| Antoni Torres Minlan Yang Ana Motos | Hospital Clinic, Barcelona |
| Carlos Luna | Hospital de Clínicas |
| Francisco Arancibia | Hospital del Tórax |
| Virginie Williams Alexandre Noel | Hospital du Sacre Coeur (Universite de Montreal) |
| Nestor Luque | Hospital Emergencia Ate Vitarte |
| Marina Fantini | Hospital Mater Dei |
| Ruth Noemi Jorge García Enrique Chicote Alvarez | Hospital Nuestra Señora de Gracia |
| Anna Greti | Hospital Puerta de Hierro |
| Adrian Ceccato | Hospital Universitari Sagrat Cor |
| Angel Sanchez | Hospital Universitario Sant Joan d’Alacant |
| Ana Loza Vazquez | Hospital Universitario Virgen de Valme |
| Ferran Roche-Campo Diego Franch-Llasat | Hospital Verge de la Cinta de Tortosa |
| Divina Tuazon | Houston Methodist Hospital |
| Marcelo Amato Luciana Cassimiro Flavio Pola Francis Ribeiro Guilherme Fonseca | INCOR (Universidade de São Paulo) |
| Heidi Dalton Mehul Desai Erik Osborn Hala Deeb | INOVA Fairfax Hospital |
| Antonio Arcadipane Gennaro Martucci Giovanna Panarello Stefano Vitiello Claudia Bianco Giovanna Occhipinti Matteo Rossetti Raffaele Cuffaro | ISMETT |
| Nidhal Siddig | Jabra Hospital, Khartoum |
| Sung-Min Cho  Glenn Whitman | Johns Hopkins |
| Marwan El Sayed Walaa Mokhtar Eslam El-Shenawy | Kafr Elsheikh University Hospital |
| Hiroaki Shimizu Naoki Moriyama | Kakogawa Acute Care Medical Center |
| Jae-Burm Kim | Keimyung University Dong San Hospital |
| Nobuya Kitamura | Kimitsu Chuo Hospital |
| Johannes Gebauer | Klinikum Passau |
| Toshiki Yokoyama | Kouritu Tousei Hospital |
| Abdulrahman Al-Fares Sarah Buabbas Esam Alamad Fatma Alawadhi Kalthoum Alawadi  Mohamed Ahmed Khalefa  Nourah Ahmad Abdulaziz Al Ajeel  Mohammad Fathy Aly  Abdullah Al-Saleh  Abdullah Naanouh  Alaa Mohammed Elshourbgy  Abdulrahman Al-Fares  Mohamed Yousef Gad  Rania Mohamed ElRazaz  Ibrahim Khadadah  Ahmed Mohammed Almumin  Hala Altarakma  Hasan Albannay  Mohammed Kh Alsaleh  Mahmoud Saad Abdallah Radwan  Islam Ahmed Saadallah | Al-Amiri and Jaber Al-Ahmed Hospitals, Kuwait Extracorporeal Life Support Program |
| Hiro Tanaka | Kyoto Medical Centre |
| Satoru Hashimoto Masaki Yamazaki | Kyoto Prefectural University of Medicine |
| Tak-Hyuck Oh | Kyung Pook National University Chilgok Hospital |
| Mark Epler Cathleen Forney  Louise Kruse Jared Feister Joelle Williamson Katherine Grobengieser | Lancaster General Health |
| Eric Gnall Sasha Golden Mara Caroline  Timothy Shapiro Colleen Karaj Lisa Thome Lynn Sher Mark Vanderland Mary Welch Sherry McDermott | Lankenau Institute of Medical Research (Main Line Health) |
| Matthew Brain Sarah Mineall  Maria Unwin  Lixian Chen  Tarnya Trezise  Laurie McKeon | Launceston General Hospital |
| Dai Kimura | Le Bonheur Children’s Hospital |
| Luca Brazzi Gabriele Sales  Giorgia Montrucchio | Le Molinette Hospital (Ospedale Molinette Torino) |
| Tawnya Ogston | Legacy Emanuel Medical Center |
| Dave Nagpal Karlee Fischer | London Health Sciences Centre |
| Roberto Lorusso  Bas van Bussell  Maria Elena De Piero  Silvia Mariani | Maastricht University Medical Centre |
| Dr Rajavardhan Rangappa Dr Rajesh Mohan Shetty  Sujin Rai P  Argin Ganesan | Manipal Hospital Whitefield |
| Samar Tharwat | Mansoura University Hospital |
| Mariano Esperatti  Nora Angélica Fuentes  Maria Eugenia Gonzalez | Hospital Privado de Comunidad. Mar del Plata. Escuela Superior de Medicina. Universidad Nacional de  Mar del Plata |
| Diarmuid O’Briain | Maroondah Hospital |
| Edmund G. Carton | Mater Misericordiae University Hospital |
| Ayan Sen Amanda Palacios Deborah Rainey | Mayo Clinic College of Medicine |
| Gordan Samoukoviv Josie Campisi | McGill University Health Centre |
| Lucia Durham Emily Neumann Cassandra Seefeldt Octavio Falcucci Amanda Emmrich Jennifer Guy Carling Johns Kelly Potzner Catherine Zimmermann Angelia Espinal | Medical College of Wisconsin (Froedtert Hospital) |
| Nina Buchtele Michael Schwameis  Andrea Korhnfehl  Roman Brock  Thomas Staudinger | Medical University of Vienna |
| Stephanie-Susanne Stecher Michaela Barnikel  Sófia Antón  Alexandra Pawlikowski | Medical Department II, LMU Hospital Munich |
| Akram Zaaqoq Lan Anh Galloway Caitlin Merley | MedStar Washington Hospital Centre |
| Mohamed Muftah | Misurata Medical Center |
| Alistair Nichol | Monash University |
| Marc Csete Luisa Quesada Isabela Saba | Mount Sinai Medical Centre |
| Daisuke Kasugai Hiroaki Hiraiwa Taku Tanaka | Nagoya University Hospital |
| Eva Marwali Yoel Purnama Santi Rahayu Dewayanti Ardiyan Dafsah Arifa Juzar Debby Siagian | National Cardiovascular Center Harapan Kita, Jakarta, Indonesia |
| Yih-Sharng Chen | National Taiwan University Hospital |
| Amer Aldhalia | Nasar City Hospital for Insurance |
| Mark Ogino | Nemours Alfred I duPont Hospital for Children |
| Prashant Nasa Christina Matthew Nimisha Abdul Majeed | NMC Al Nahda Hospital Dubai |
| Wael Hafez | NMC Royal Hospital, Abu Dhabi |
| Indrek Ratsep Andra-Maris Post Piret Sillaots  Anneli Krund  Merili-Helen Lehiste  Tanel Lepik | North Estonia Medical Centre |
| Frank Manetta Effe Mihelis Iam Claire Sarmiento Mangala Narasimhan Michael Varrone | Northwell Health |
| Mamoru Komats | Obihiro-Kosei General Hospital |
| Julia Garcia-Diaz Catherine Harmon | Ochsner Clinic Foundation |
| S. Veena Satyapriya Amar Bhatt Nahush A. Mokadam Alberto Uribe Alicia Gonzalez Haixia Shi Johnny McKeown Joshua Pasek Juan Fiorda Marco Echeverria | Ohio State University Medical Centre |
| Rita Moreno | Oklahoma Heart Institute |
| Bishoy Zakhary | Oregon Health and Science University Hospital (OHSU) |
| Marco Cavana Alberto Cucino | Ospedale di Arco (Trento Hospital) |
| Giuseppe Foti Marco Giani Benedetta Fumagalli | Ospedale San Gerardo |
| Davide Chiumello Valentina Castagna | Ospedale San Paolo |
| Andrea Dell’Amore Paolo Navalesi | Padua University Hospital (Policlinico of Padova) |
| Hoi-Ping Shum | Pamela Youde Nethersole Eastern Hospital |
| Alain Vuysteke | Papworth Hospitals NHS Foundation Trust |
| Asad Usman Andrew Acker Benjamin Smood Blake Mergler Federico Sertic Madhu Subramanian Alexandra Sperry Nicolas Rizer | Penn Medicine (Hospital of the University of Pennsylvania) |
| Erlina Burhan  Menaldi Rasmin Ernita Akmal Faya Sitompul Navy Lolong Bhat Naivedh | Persahabatan General Hospital |
| Simon Erickson | Perth Children's Hospital |
| Peter Barrett David Dean Julia Daugherty | Piedmont Atlanta Hospital |
| Antonio Loforte | Policlinico di S. Orsola, Università di Bologna |
| Irfan Khan Mohammed Abraar Quraishi Olivia DeSantis | Presbyterian Hospital Services, Albuquerque |
| Ahmad Nasrallah | Prince Hamza Hospital- Amman |
| Dominic So Darshana Kandamby | Princess Margaret Hospital |
| Jose M. Mandei Hans Natanael | Prof Dr R. D. Kandou General Hospital - Paediatric |
| Eka YudhaLantang Anastasia Lantang | Prof Dr R. D R. D. Kandou General Hospital - Adult |
| Surya Oto Wijaya | Dr Sulianti Saroso Hospital |
| Anna Jung | Providence Saint John's Health Centre |
| George Ng Wing Yiu Ng | Queen Elizabeth Hospital, Hong Kong |
| Pauline Yeung Ng  Shu Fang | The University of Hong Kong |
| Alexis Tabah Megan Ratcliffe Maree Duroux | Redcliffe Hospital |
| Ahmed Alajeeli  Ali Tarhabat | Regdalin Hospital |
| Shingo Adachi Shota Nakao | Rinku General Medical Center (and Senshu Trauma and Critical Care Center) |
| Pablo Blanco Ana Prieto Jesús Sánchez | Rio Hortega University Hospital |
| Meghan Nicholson | Rochester General Hospital |
| Warwick Butt Alyssa Serratore Carmel Delzoppo | Royal Children’s Hospital |
| Pierre Janin Elizabeth Yarad | Royal North Shore Hospital |
| Richard Totaro Jennifer Coles | Royal Prince Alfred Hospital |
| Bambang Pujo | RSUD Soetomo |
| Robert Balk Andy Vissing Esha Kapania James Hays Samuel Fox Garrett Yantosh Pavel Mishin | Rush University, Chicago |
| Safia Adem | Sabha Medical Center |
| Saptadi Yuliarto Kohar Hari Santoso Susanthy Djajalaksana | Saiful Anwar Malang Hospital (Brawijaya University) (Paediatrics) |
| Arie Zainul Fatoni | Saiful Anwar Malang Hospital (Brawijaya University) (Adult) |
| Masahiro Fukuda | Saiseikai Senri Hospital |
| Keibun Liu | Saiseikai Utsunomiya Hospital |
| Paolo Pelosi Denise Battaglini  Chiara Robba | San Martino Hospital |
| Juan Fernando Masa Jiménez | San Pedro de Alcantara Hospital |
| Diego Bastos | Sao Camilo Cura D’ars |
| Sérgio Gaião | São João Hospital Centre, Porto |
| Desy Rusmawatiningtyas | Sardjito Hospital (Paediatrics) |
| Young-Jae Cho | Seoul National University Bundang Hospital |
| Su Hwan Lee | Severance Hospital |
| Tatsuya Kawasaki | Shizuoka Children’s Hospital |
| Laveena Munshi | Sinai Health Systems (Mount Sinai Hospital) |
| Pranya Sakiyalak Prompak Nitayavardhana | Siriraj Hospital |
| Mohamed Bashir Elagili  Talat Ahmed Abu Salem | Soug Althulatha Isolation Center |
| Tamara Seitz | Sozialmedizinisches Zentrum Süd – Kaiser-Franz-Josef-Spital |
| Rakesh Arora David Kent | St Boniface Hospital (University of Mannitoba) |
| Daniel Marino | St Christopher’s Hospital for Children |
| Swapnil Parwar Andrew Cheng Jennene Miller | St George Hospital |
| Shigeki Fujitani Naoki Shimizu | St Marianna Medical University Hospital |
| Jai Madhok Clark Owyang | Stanford University Hospital |
| Hergen Buscher Claire Reynolds | St Vincent’s Hospital |
| Abusalama Abdurraouf  Ali Abdulnasir Kredan  Abdurrahman Haddud | Swani Health Isolation Center |
| Saad Moharam | Tanta University Hospital |
| Olavi Maasikas AleksanBeljantsev Vladislav Mihnovits | Tartu University Hospital |
| Takako Akimoto Mariko Aizawa Kanako Horibe Ryota Onodera | Teine Keijinkai Hospital |
| Carol Hodgson Aidan Burrell Meredith Young | The Alfred Hospital |
| Timothy George | The Heart Hospital Baylor Plano, Plano |
| Kiran Shekar  Niki McGuinness Lacey Irvine | The Prince Charles Hospital |
| Brigid Flynn | The University of Kansas Medical Centre |
| Abdulrahman Almjersah  Ashraf Bakri | Tishreen University Hospital |
| Tomoyuki Endo | Tohoku Medical and Pharmaceutical University |
| Kazuhiro Sugiyama | Tokyo Metropolitan Bokutoh Hospital |
| Keiki Shimizu | Tokyo Metropolitan Medical Center |
| Eddy Fan Kathleen Exconde | Toronto General Hospital |
| Shingo Ichiba | Tokyo Women’s Medical University Hospital |
| Muhannud Binnawara | Tripoli Central Hospital |
| Hussein Embarek | Tripoli University Hospital |
| Leslie Lussier | Tufts Medical Centre (and Floating Hospital for Children) |
| Gösta Lotz | Universitätsklinikum Frankfurt (University Hospital Frankfurt) (Uniklinik) |
| Maximilian Malfertheiner Lars Maier Esther Dreier | Universitätsklinikum Regensburg (Klinik für Innere Medizin II) |
| Neurinda Permata Kusumastuti | University Airlangga Hospital (Paediatric) |
| Colin McCloskey Al-Awwab Dabaliz Tarek B Elshazly Josiah Smith | University Hospital Cleveland Medical Centre (UH Cleveland Hospital) |
| Konstanty S. Szuldrzynski Piotr Bielański | University Hospital in Krakow |
| Yusuff Hakeem | University Hospitals of Leicester NHS Trust (Glenfield Hospital) |
| Keith Wille | University of Alabama at Birmingham Hospital (UAB) |
| Srinivas Murthy | University of British Columbia |
| Ken Kuljit S. Parhar Kirsten M. Fiest  Cassidy Codan Anmol Shahid | University of Calgary (Peter Lougheed Centre, Foothills Medical Centre, South Health Campus and Rockyview General Hospital) |
| Mohamed Fayed Timothy Evans Rebekah Garcia Ashley Gutierrez Hiroaki Shimizu | University of California, San Francisco-Fresno Clinical Research Centre |
| Tae Song Rebecca Rose | University of Chicago |
| Suzanne Bennett Denise Richardson | University of Cincinnati Medical Centre |
| Giles Peek | University of Florida |
| Lovkesh Arora Kristina Rappapport Kristina Rudolph Zita Sibenaller Lori Stout Alicia Walter | University of Iowa |
| Daniel Herr Nazli Vedadi | University of Maryland - Baltimore |
| Robert Bartlett | University of Michigan Medical Center |
| Antonio Pesenti | University of Milan |
| Shaun Thompson Julie Hoffman Xiaonan Ying Bailey Williams Emely Sanchez Chika Akwani | University of Nebraska Medical Centre |
| Ryan Kennedy | University of Oklahoma Health Sciences Centre (OU) |
| Muhammed Elhadi | Faculty of Medicine, University of Tripoli |
| Matthew Griffee  Mary Mone Anna Ciullo Yuri Kida | University of Utah Hospital |
| Ricard Ferrer Roca JordI Riera Sofia Contreras Cynthia Alegre | Vall d'Hebron University Hospital, Barcelona |
| Christy Kay Irene Fischer Elizabeth Renner | Washington University in St. Louis/ Barnes Jewish Hospital |
| Hayato Taniguci | Yokohama City University Medical Center |
| James Lee Daniel Plotkin Barbara Wanjiru Citarella Laura Merson | ISARIC, Centre for Tropical Medicine and Global Health, University of Oxford, Oxford, UK |

**Collaborators**

| **Prefix/First Name/Last Name** | **Site Name** |
| --- | --- |
| Emma Hartley | Aberdeen Royal Infirmary (Foresterhill Health Campus) |
| Bastian Lubis | Adam Malik Hospital |
| Takanari Ikeyama | Aichi Childrens Health and Medical Center |
| Alshaymaa Mortada | Ain Shams University |
| Ameen Alhamad | Aleppo University Hospital |
| Ahmed Mechi | Al-Sader Teaching Hospital, Al-Najaf |
| Islam Mohsen Ali Mohamed Hassan Nadar | Al Salam Specialized Hospital |
| Mohammed Saleh Alyasiri | Alshifa Center Medical City |
| Muhammed Zainab Alghali Elsaid | Alshuhada Hospital |
| Balu Bhaskar | American Hospital |
| Jae-Seung Jung | Anam Korea University Hospital |
| Shay McGuinness | Auckland City Hospital |
| Glenn Eastwood | Austin Hospital |
| Sandra Rossi Marta  Fabio Guarracino | Azienda Ospedaliero Universitaria Parma |
| Stacy Gerle | Banner University Medical Centre |
| Emily Coxon | Baptist Health Louisville |
| Bruno Claro | Barts Hospital |
| Wafa Aldressi | Benghazi Medical Centre |
| Mahmoud Eleisawy | Benha University Hospital |
| Hasnaa Osama | Beni-Suef University Hospital |
| Daniel Loverde | Billings Clinic |
| Namrata Patil | Brigham and Women’s Hospital |
| Vieri Parrini | Borgo San Lorenzo Hospital |
| Angela McBride | Brighton and Sussex Medical School |
| Kathryn Negaard | Brooke Army Medical Centre |
| Angela Ratsch | Bundaberg Hospital |
| Ahmad Abdelaziz | Cairo University Hospital |
| Juan David Uribe | Cardio VID |
| Adriano Peris | Careggi Hospital |
| Mark Sanders | Cedar Park Regional Medical Center |
| Dominic Emerson | Cedars-Sinai Medical Centre |
| Muhammad Kamal | Cengkareng Hospital |
| Hamza Faida | Centre Hospitalier Universitaire Ibn Sina Rabat |
| Pedro Povoa | Centro Hospitalar de Lisboa |
| Roland Francis | Charite-Univerrsitatsmedizi n Berlin |
| Ali Cherif | Charles Nicolle University Hospital |
| Sunimol Joseph | Children’s Health Ireland (CHI) at Crumlin |
| Matteo Di Nardo | Children’s Hospital Bambino Gesù |
| Micheal Heard | Children's Healthcare of Atlanta – Egleston Hospital |
| Kimberly Kyle | Children's Hospital – Los Angeles |
| Ray A Blackwell | Christiana Care Health System's Centre for Heart and Vascular Health |
| Amel OUYAHIA | CHU - Chu Saadna Abdenour De Sétif - Sétif |
| Michael Piagnerelli  Patrick Biston | CHU de Charleroi |
| Hye Won Jeong | Chungbuk National University Hospital |
| Reanna Smith | Cincinnati Children's |
| Yogi Prawira | Cipto Mangunkusumo Hospital |
| Giorgia Montrucchio | Città della Salute e della Scienza Hospital – Turin, Italy |
| Arturo Huerta Garcia | Clínica Sagrada Família |
| Nahikari Salterain | Clinica Universidad de Navarra |
| Bart Meyns | Collaborative Centre Department Cardiac Surgery, UZ Leuven |
| Muhammed Elnasser | Damascus Hospital |
| Marsha Moreno | Dignity Health Medical Group- Dominican |
| Rajat Walia | Dignity Health St. Joseph's Hospital and Medical Center (SJHMC) |
| Amit Mehta | Doernbecher Children’s Hospital |
| Annette Schweda | Donaustauf Hospital |
| Melissa Williams | Duke University Hospital (Durham) |
| Emad Amkhatirah | Elmarj Teaching Hospital |
| Kyung Hoon Kim | Eunpyeung St Mary's Hospital |
| Alexandra Assad | Fluminense Federal University |
| Estefania Giraldo | Fundación Clinica Shaio (Shaio Clinic) |
| Wojtek Karolak | Gdansk Medical University |
| Martin Balik | General University Hospital |
| Elizabeth Pocock | George Washington University Hospital |
| Akram Mohamed | Gharyan Central Hospital |
| Evan Gajkowski | Giesinger Medical Centre |
| Mohamed Bedair | Giza International Hospital |
| Kanamoto Masafumi | Gunma University Graduate School of Medicine |
| Nicholas Barrett | Guy's and St Thomas NHS Foundation Trust Hospital |
| Yoshihiro Takeyama | Hakodate City Hospital |
| Sunghoon Park | Hallym University Sacred Heart Hospital |
| Faizan Amin | Hamilton General Hospital |
| Fina Meilyana Andriyani | Hasan Sadikin Hospital (Paediatric) |
| Serhii Sudakevych | Heart Institute Ministry of Health of Ukraine |
| Janos Schnur | Heim Pál National Pediatric Institute |
| Angela Ratsch | Hervey Bay Hospital |
| Magdalena Vera | Hospital Clinico de la Pontificia Universidad Catolica |
| Rodrigo Cornejo | Hospital Clinico de la Universidad de Chile |
| Patrícia Schwarz  Ana Carolina Mardini | Hospital de Clínicas de Porto Alegre |
| Thais de Paula | Hospital Felicio Rocho |
| Ary Serpa Neto | Hospital Israelita Albert Einstein |
| Andrea Villoldo | Hospital Privado de Comunidad |
| Alexandre Siciliano Colafranceschi | Hospital Pro Cardíaco |
| Alejandro Ubeda Iglesias | Hospital Punta de Europa |
| Juan Granjean | Hospital Regional de Valdivia |
| Lívia Maria Garcia Melro  Giovana Fioravante Romualdo | Hospital Samaritano Paulista |
| Diego Gaia | Hospital Santa Catarina |
| Helmgton Souza | Hospital Santa Marta |
| Filomena Galas | Hospital Sirio Libanes |
| Rafael Máñez Mendiluce | Hospital Universitario de Bellvitge |
| Alejandra Sosa | Hospital Universitario Esperanza (Universidad Francisco Marroquin) |
| Ignacio Martinez | Hospital Universitario Lucus Augusti |
| Hiroshi Kurosawa | Hyogo Prefectural Kobe Children's Hospital |
| Mohammad Badr Almoshantaf | Ibn Al-Nafees Hospital |
| Juan Salgado | Indiana University Health |
| Beate Hugi-Mayr | Inselspital University Hospital |
| Eric Charbonneau | Institut Universitaire de Cardiologie et de Pneumologie de Quebec - Universite Laval |
| Vitor Salvatore Barzilai | Instituto de Cardiologia do Distrito Federal - ICDF |
| Veronica Monteiro | Instituto de Medicina Integral . Fernando Figueira (IMIP) |
| Rodrigo Ribeiro de Souza | Instituto Goiano de Diagnostico Cardiovascular (IGDC) |
| Michael Harper | INTEGRIS Baptist Medical Center |
| Hiroyuki Suzuki | Japan Red Cross Maebashi Hospital |
| Celina Adams | John C Lincoln Medical Centre |
| Jorge Brieva | John Hunter Hospital |
| Almu'atasim Khamees | Jordan University Hospital |
| Fadi Graige | Kalamoon Hospital |
| Moh Supriatna | Kariadi Hospital Semarang |
| George Nyale | Kenyatta National Hospital (KNH) |
| Faisal Saleem Eltatar | King Abdullah Medical City |
| Jihan Fatani | King Abdullah Medical City Specialist Hospital |
| Husam Baeissa | King Abdullah Medical Complex |
| Ayman AL Masri | King Salman Hospital NWAF |
| Ahmed Rabie | King Saud Medical City |
| Mok Yee Hui | KK Women's and Children's Hospital |
| Masahiro Yamane | KKR Medical Center |
| Hanna Jung | Kyung Pook National University Hospital |
| Ayorinde Mojisola Margaret | Lagos University Teaching Hospital |
| Newell Nacpil | Lung Center of the Philippines |
| Katja Ruck | Luxembourg Heart Center |
| Rhonda Bakken | M Health Fairview |
| Claire Jara | Maine Medical Centre (Portland Maine) |
| Tim Felton | Manchester University NHS Foundation Trust - Wythenshawe |
| Lorenzo Berra | Massachusetts General Hospital |
| Bobby Shah | Medanta Hospital |
| Arpan Chakraborty | Medica Super speciality Hospital |
| Monika Cardona | Medical University of South Carolina |
| Gerry Capatos | Mediclinic Parkview Hospital Dubai |
| Bindu Akkanti | Memorial Hermann - Texas Medical Centre |
| Abiodun Orija | Memorial Regional Hospital (Hollywood Florida) |
| Harsh Jain | Mercy Hospital of Buffalo |
| Asami Ito | Mie University Hospital |
| Brahim Housni | Mohammed VI University Hospital |
| Sennen Low | National Centre for Infectious Diseases |
| Koji Iihara | National Cerebral and Cardiovascular Center |
| Joselito Chavez | National Kidney and Transplant Institute |
| Kollengode Ramanathan | National University Hospital, Singapore |
| Gustavo Zabert | National University of Comahue |
| Krubin Naidoo | Nelson Mandela Children's Hospital |
| Ian Seppelt | Nepean Hospital |
| Marlice VanDyk  Sarah MacDonald | Netcare Unitas ECMO Centre |
| Shingo Ichiba | Nippon Medical School Hospital |
| Randy McGregor | Northwestern Medicine |
| Teka Siebenaler | Norton Children's Hospital |
| Hannah Flynn | Novant Health (NH) Presbyterian Medical Centre |
| Kristi Lofton | Ochsner LSA Health Shreveport |
| Toshiyuki Aokage | Okayama University Hospital |
| Bakar Kvirkvelia | Open Heart 5^th^ Clinical Hospital, Tbilisi |
| Kazuaki Shigemitsu | Osaka City General Hospital |
| Andrea Moscatelli | Ospedale Gaslini |
| Giuseppe Fiorentino | Ospedali dei Colli |
| Matthias Baumgaertel | Paracelsus Medical University Nuremberg |
| Serge Eddy Mba | Parirenyatwa General Hospital |
| Jana Assy | Pediatric and Neonatal Cardiac Intensive Care at the American University |
| Amelya Hutahaean | Pelni Hospital |
| Holly Roush | Penn State Heath S. Hershey Medical Centre |
| Kay A Sichting | Peyton Manning Children's Hospital |
| Francesco Alessandri | Policlinico Umberto, Sapienza University of Rome |
| Debra Burns | Presbyterian Hospital, New York/ Weill Cornell Medical Centre |
| Taha Husayn Alkhubouli | Preventive Medicine Hospital |
| Ahmad Nasrallah | Prince Hamza Hospital- Amman |
| Ahmed Rabie | Prince Mohammed bin Abdulaziz Hospital |
| Gavin Salt | Prince of Wales |
| Carl P. Garabedian | Providence Sacred Heart Children's Hospital |
| Jonathan Millar  Malcolm Sim | Queen Elizabeth II University Hospital |
| Adrian Mattke | Queensland Children’s Hospital |
| Danny McAuley | Queens University of Belfast |
| Jawad Tadili | Rabat University Hospital |
| Tim Frenzel | Radboud University Medical Centre |
| Amro Abuleil | Rafidia Surgical Hospital |
| Yaron Bar-Lavie | Rambam Hospital |
| Aaron Blandino Ortiz | Ramón y Cajal University Hospital |
| Jackie Stone | Rapha Medical Centre |
| Alexis Tabah | Redcliffe Hospital |
| Antony Attokaran | Rockhampton Hospital |
| Michael Farquharson | Royal Adelaide Hospital |
| Brij Patel | Royal Brompton & Harefield NHS Foundation Trust |
| Derek Gunning | Royal Columbian Hospital |
| Kenneth Baillie | Royal Infirmary Edinburgh |
| Pia Watson | Sahlgrenska University Hospital |
| Kenji Tamai | Saiseikai Yokohamashi Tobu Hospital |
| Gede Ketut Sajinadiyasa  Dyah Kanyawati | Sanglah General Hospital |
| Marcello Salgado | Santa Casa de Misericordia de Juiz de Fora |
| Assad Sassine | Santa Casa de Misericórdia de Vitoria |
| Bhirowo Yudo | Sardjito Hospital |
| Scott McCaul | Scripps Memorial Hospital La Jolla |
| Bongjin Lee | Seoul National University Children's Hospital |
| Sang Min Lee | Seoul National University Hospital |
| Arnon Afek | Sheba Medical Center |
| Shimaa E Fattouh | Sherbin General Hospital |
| Yoshiaki Iwashita | Shimane University Hospital |
| Hammad Fadlalmola | Soba University Hospital |
| Bambang Pujo Semedi  Neurinda Permata Kusumastuti | Soetomo General Hospital (FK UNAIR) |
| Noureldin Mohamed Mansour | Souad Kafafi University Hospital |
| Jack Metiva | Spectrum Health Western Governors University |
| Nicole Van Belle | St. Antonius Hospital |
| Ignacio Martin-Loeches | St James’s University Hospital |
| Dr Mohammed Al-Sadawi | Stony Brook University |
| Cenk Kirakli | Suat Seren Chest Diseases and Surgery Practice and Training Centre |
| Al-Touny Shimaa | Suez Canal University Hospitals |
| Lenny Ivatt | Swansea Hospital |
| Chia Yew Woon | Tan Tock Seng Hospital |
| Hyun Mi Kang | The Catholic University of Seoul St Mary Hospital |
| Timothy Smith | The Christ Hospital |
| Erskine James | The Medical Centre Navicent Health |
| Nawar Al-Rawas | Thomas Jefferson University Hospital |
| Yudai Iwasaki | Tohoku University |
| Hamza Ashour | Traghen Hospital |
| Kenny Chan King-Chung | Tuen Mun Hospital |
| Vadim Gudzenko | UCLA Medical Centre (Ronald Regan) |
| Beate Hugi-Mayr | Universitätsspital Bern, Universitätsklinik für Herz- und Gefässchirurgie |
| Fabio Taccone | Universite Libre de Bruxelles |
| Fajar Perdhana | University Airlangga Hospital (Adult) |
| Yoan Lamarche | University de Montreal (Montreal Heart Institute) |
| Joao Miguel Ribeiro | University Hospital CHLN |
| Nikola Bradic | University Hospital Dubrava |
| Klaartje Van den Bossche | University Hospital Leuven |
| Oude Lansink | University Medical Center Groningen |
| Gurmeet Singh | University of Aberta (Mazankowski Heart Institute) |
| Gerdy Debeuckelaere | University of Antwerp |
| Henry T. Stelfox | University of Calgary and Alberta Health Services |
| Cassia Yi | University of California at San Diego |
| Jennifer Elia | University of California, Irvine |
| Thomas Tribble | University of Kentucky Medical Center |
| Shyam Shankar | University of Missouri |
| Raj Padmanabhan | University of Pittsburgh Medical Centre |
| Bill Hallinan | University of Rochester Medical Centre (UR Medicine) |
| Luca Paoletti | University of South Carolina |
| Yolanda Leyva | University of Texas Medical Branch |
| Tatuma Fykuda | University of the Ryukyus |
| Jenelle Badulak | University of Washington in Seattle |
| Jillian Koch | University of Wisconsin & American Family Children's Hospital |
| Lisa Janowaik | UTHealth (University of Texas) |
| Amy Hackman | UT Southwestern |
| Deb Hernandez | Valley Children's Hospital (Madera) |
| Jennifer Osofsky | Vassar Brothers Medical Center (VBMC) |
| Katia Donadello | Verona Integrated University Hospital |
| Aizah Lawang | Wahidin Sudirohusodo Hospital |
| Josh Fine | WellSpan Health - York Hospital |
| Benjamin Davidson | Westmead Hospital |
| Andres Oswaldo Razo Vazquez | Yale New Haven Hospital |
| Ibrahim Abdehaleem | Zagazig University Hospital |
